# Searching for Suitable Kojic Acid Coformers: From Cocrystals and Salt to Eutectics

**DOI:** 10.1021/acs.cgd.2c01364

**Published:** 2023-02-09

**Authors:** Renren Sun, Doris E. Braun, Lucia Casali, Dario Braga, Fabrizia Grepioni

**Affiliations:** † Department of Chemistry “G. Ciamician”, University of Bologna, Via Selmi 2, 40126 Bologna, Italy; ‡ School of Chemical Engineering, Zhengzhou University, 450001 Zhengzou, Henan Province, The People’s Republic of China; § Institute of Pharmacy, University of Innsbruck, Innrain 52c, 6020 Innsbruck, Austria

## Abstract

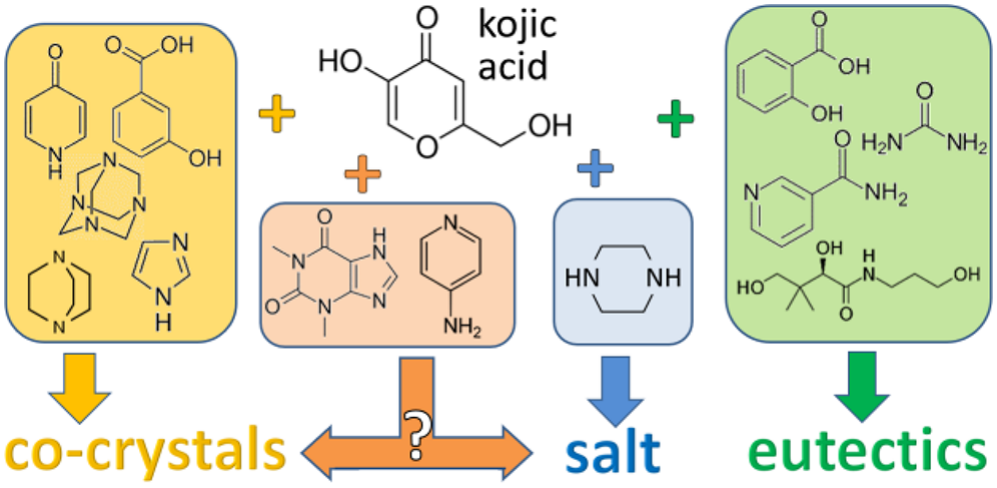

The possibility of obtaining cocrystals of kojic acid with organic coformers has been investigated by both computational and experimental approaches. Cocrystallization attempts have been carried out with about 50 coformers, in different stoichiometric ratios, by solution, slurry, and mechanochemical methods. Cocrystals were obtained with 3-hydroxybenzoic acid, imidazole, 4-pyridone, DABCO, and urotropine, while piperazine yielded a salt with the kojiate anion; cocrystallization with theophylline and 4-aminopyridine resulted in stoichiometric crystalline complexes that could not be described with certainty as cocrystals or salts. In the cases of panthenol, nicotinamide, urea, and salicylic acid the eutectic systems with kojic acid were investigated via differential scanning calorimetry. In all other preparations the resulting materials were constituted of a mixture of the reactants. All compounds were investigated by powder X-ray diffraction; the five cocrystals and the salt were fully characterized via single crystal X-ray diffraction. The stability of the cocrystals and the intermolecular interactions in all characterized compounds have been investigated by computational methods based on the electronic structure and pairwise energy calculations, respectively.

## Abstract 2

Cocrystals of kojic acid, not easily prone to cocrystal formation, and salt(s) were obtained by slurry, mechanochemistry, and solution crystallization; eutectic mixtures were also explored. Structures and stabilities were investigated by combined computational, diffraction, and calorimetric methods.

## Introduction

Cocrystallization is one of the most fruitful applications of the crystal engineering idea,^1^ namely the purposeful supramolecular aggregation via noncovalent interactions of molecules in crystals, in order to obtain novel, or improved, solid state properties. As a matter of fact, cocrystallization of molecular and/or ionic components, each forming stable solids at room temperature, is being actively investigated in many different areas such as pharmaceutics,^2^ agrochemistry,^3^ food,^4,5^ and high-energy materials^6^ with the aim of preparing multicomponent supramolecular aggregates with new or diverse collective solid state physical and chemical properties. In the pharmaceutical field, in particular, cocrystallization with pharmaceutically acceptable coformers is extensively explored to improve fundamental properties of the active pharmaceutical ingredients (API) such as the solubility and dissolution rate, but also thermal stability and processability. In addition to this, the API can also be designed as a codrug by cocrystallization with another active ingredient. Then, not only do the solid-state physicochemical properties of APIs change relative to those of parent crystals, but biological activity may also yield significantly different results.

**Scheme 1:**
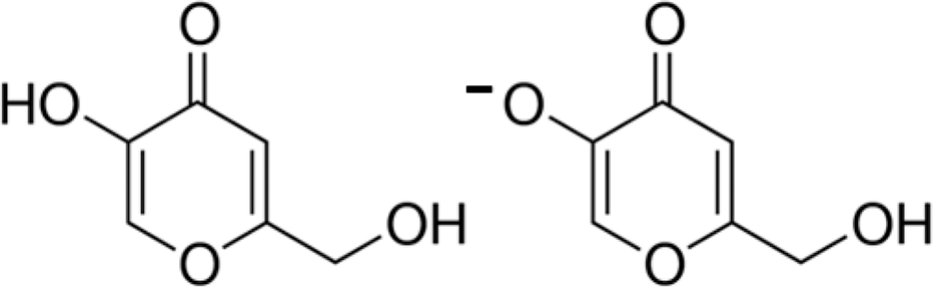
Kojic Acid (HKA, left) and the Kojiate Anion (KA^–^, right)

In this paper we explore the cocrystallization of the mild antibacterial agent kojic acid^7^ (HKA, shown in Scheme 1) with suitable coformers.

Kojic acid (HKA) is a natural fungal metabolite, first isolated from *Aspergillus oryzae*. HKA and its derivatives are used as whitening agents in the cosmetic industry, as well as antiseptic and moisturizing agents.^8,9^ Kojic acid can be used as a food additive for preservation, with antiseptic and antioxidant effects. HKA and its derivatives are widely used as antibacterial agents toward many types of bacteria, such as *Escherichia coli* and *Staphylococcus aureus*.^10^

Indeed, the motivation for this study stems from the need to fight the emerging threat of antimicrobial resistance (AMR). AMR is becoming one of the major medical challenges in most healthcare systems, and refers to the ability of microorganisms to develop resistance to the action of originally efficacious antibiotics; that is, the sensitivity to drugs is reduced.^11,12^

In this work we explore the possibility of preparing a series of cocrystals of kojic acid with a number of selected coformers to enrich the library of novel materials with potential antibacterial properties. In order to select adequate coformers, we are guided by the information available in the literature on the antibacterial properties of molecules such as imidazoles,^13^ piperazine,^14^ 3-hydroxybenzoic acid,^15^ and urotropine.^16^ Furthermore, it is known that 4-pyridine and 1,4-diazabicyclo[2.2.2]octane (DABCO) are effective antioxidants. 4-Pyridine is also an important raw material for the synthesis of antibacterial active pyridine quaternary ammonium salts,^17^ and the DABCO bis-quaternary salts also exhibit antibacterial activity.^18^ Theophylline has also been shown to possess antiaging and anticytotoxic effects in human skin.^19^ Caffeine is highly biologically active, has the ability to penetrate the skin barrier, and also prevents excessive accumulation of fat in skin cells.^20^ Theobromine can scavenge reactive oxygen species (ROS) produced in the skin after UV exposure.^21,22^ Nicotinamide is also widely used as a cosmetic skin care ingredient for its whitening, antiaging, and skin barrier building properties.^23,24^ The list was further extended to comprise 4-aminopyridine, which is also known to enhance the release of presynaptic acetylcholine and increase the force of muscle contractions, thus behaving as an antagonist of the neuromuscular blockade effects of antibiotics.^25,26^

Beside organic compounds already known to possess mild antimicrobial activity, a number of other cocrystallization partners have been selected by using data mining tools available within the Cambridge Structural Database,^27^ and their stability evaluated by available computational methods.

The coformers that have yielded novel materials by solution, slurry, and mechanochemical methods, whether as cocrystals, salts, or eutectic compositions by cocrystallization with kojic acid, are listed in Table 1. As we will discuss in the following, we were able to obtain HKA cocrystals with 3-HBA, imidazole, 4-pyridone, DABCO and urotropine, while piperazine formed a salt; in the case of theophylline and 4-aminopyridine a stoichiometric complex was also obtained, although its cocrystal/salt nature could not be determined with certainty (see Results and Discussion). The eutectic systems formed by HKA with panthenol, nicotinamide, urea, and salicylic acid have also been explored, as described in a further section. The products of successful preparations shown in Table 1 have all been characterized by calorimetric and diffraction methods.

**Table 1:**
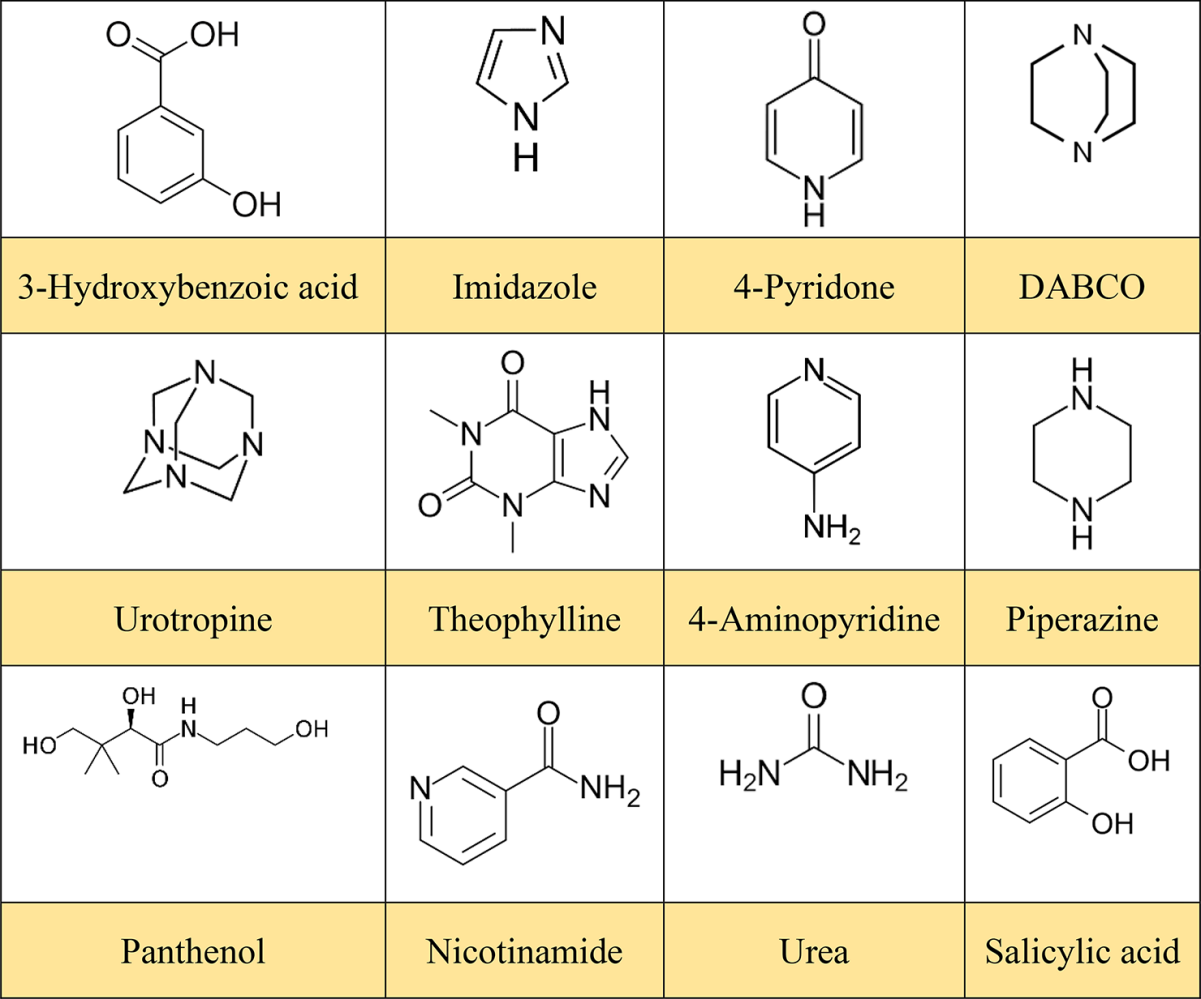
Molecules Selected as HKA Co-Formers for the Preparation of the Solid Systems Described in This Work

## Experimental Section

### Materials

Kojic acid was purchased from TCI. 3-Hydroxybenzoic acid (3HBA), imidazole, 4-pyridone, DABCO, urotropine, theophylline, piperazine (PIP), panthenol, nicotinamide, urea, salicylic acid (SA), 4-aminopyridine, and solvents were purchased from Sigma-Aldrich. Distilled water was used. All compounds were used without further purification.

### Mechanochemical Synthesis

The cocrystals HKA·imidazole, HKA·4-pyridone, and the complex HKA·4-aminopyridine were obtained by ball milling equimolar quantities of HKA (0.5 mmol) and coformer (0.5 mmol), in a 5 mL agate jar in the presence of two drops (100 μL) of water, and two 3 mm agate balls, for 60 min in a Retsch MM200 ball miller, operated at a frequency of 20 Hz. [H_2_PIP][KA]_2_·2H_2_O was obtained in the same ball milling conditions as HKA·imidazole and HKA·4-pyridone, but two drops of ethanol had to be added to the reacting mixture. The complex (HKA)_4_·(theophylline)_3_ can be produced by ball milling in the presence of two drops of methanol, ethanol, acetone, ethyl acetate, or acetonitrile. HKA·DABCO was obtained by manual grinding for 10 min, with an agate mortar and pestle, and equimolar amounts of HKA (0.5 mmol) and DABCO (0.5 mmol), either in the absence of solvent or in the presence of two drops of water, ethanol, or methanol.

### Slurry Synthesis

The crystalline complex of HKA with theophylline was obtained by slurry a 4:3 stoichiometric ratio mixture of HKA (1.2 mmol) and theophylline (0.9 mmol) in 5 mL of methanol, ethanol, acetone, ethyl acetate, or acetonitrile. (HKA)_2_·urotropine was obtained by slurry a 2:1 stoichiometric ratio mixture of HKA (1.2 mmol) and urotropine (0.6 mmol) in a solution of 5 mL of ethanol and 1 mL of water.

### Solution Synthesis

Single crystals of (HKA)_2_·urotropine could be obtained via slow evaporation at ambient conditions of an aqueous solution (5 mL) containing 0.5 mmol of urotropine and 1.0 mmol of HKA. The cocrystals HKA·3HBA and HKA·4-pyridone were obtained by slow evaporation at ambient conditions of a water:ethanol 1:1 solution containing equimolar amounts of HKA (0.5 mmol) and coformer (0.5 mmol). Single crystals of HKA·DABCO and HKA·imidazole were obtained by solvent evaporation at ambient conditions of an ethanol solution containing the products of manual grinding. Single crystals of [H_2_PIP][KA]_2_·2H_2_O were recovered by slow evaporation at ambient conditions of a methanol:ethanol 1:1 solution containing equimolar amounts of HKA (0.5 mmol) and piperazine (0.5 mmol).

### Single Crystal X-ray Diffraction

Single crystal X-ray diffraction data were collected at room temperature with an Oxford Diffraction X’Calibur equipped with a graphite monochromator and a CCD detector. Mo–Kα radiation (λ = 0.71073 Å) was used. Table S1 reports data collection and refinement details. Structural solution and refinement by least-squares methods against F^2^ were carried out using SHELXT^28^ and SHELXL^29^ implemented in the Olex2^30^ software. Non-hydrogen atoms were refined anisotropically. Highest peaks of residual electron density from Fourier maps indicated, for all structures, the position of all the hydrogen atoms belonging to -NH and -OH groups, thus confirming the cocrystal/salt attribution; for sake of better refinement, these hydrogens were added in calculated positions and refined riding on their respective N or O atoms. The program Mercury^31^ was used for graphic representations and for simulation of X-ray powder patterns on the basis of single crystal data, either retrieved from the Cambridge Structural Database (CSD) or collected in this work. The program PLATON was used for calculation of intermolecular hydrogen bonds.^32^

### X-ray Diffraction from Powder

The powder X-ray diffraction (PXRD) patterns were collected on a PANalytical X’Pert Pro automated diffractometer equipped with an X’Celerator detector in Bragg–Brentano geometry, using Cu–Kα radiation (λ = 1.5418 Å), without a monochromator, in the 2θ range 5°–40° (continuous scan mode, step size 0.033°; time/step 40 s; Soller slit 0,04 rad; antiscatter slit 1/2; divergence slit 1/4; 40 mA × 40 kV).

### Variable Temperature X-ray Diffraction

X-ray powder diffractograms in the 5–40° 2θ range were collected on a PANalytical X’Pert PRO automated diffractometer equipped with an X’Celerator detector and an Anton Paar TTK 450 system for measurements at controlled temperature. The data were collected in open air in Bragg–Brentano geometry using CuKα radiation without a monochromator.

### Differential Scanning Calorimetry (DSC)

DSC traces were recorded using a PerkinElmer Diamond differential scanning calorimeter. All samples (ca. 5–10 mg) were placed in open Al-pans. All measurements were conducted under N_2_ atmosphere in the 35–200 °C temperature range, at the heating rate of 10.00/5.00/2.00 °C min^–1^.

### Thermogravimetric Analysis (TGA)

TGA measurements for the crystalline complexes (HKA)_4_·(theophylline)_3_ and HKA·4-aminopyridine were performed with a PerkinElmer TGA7 in the temperature range 40–300/500 °C under N_2_ gas flow at a heating rate of 5.00 °C min^–1^.

### Construction of Binary Phase Diagrams

HKA:Urea, HKA:Salicylic acid, HKA:Panthenol, and HKA:Nicotinamide mixtures with HKA mole fractions ranging from 0 to 1 were thoroughly ground with an agate mortar and pestle at ambient temperature for 10 min; the microcrystalline powders thus obtained were analyzed via powder X-ray diffraction and differential scanning calorimetry (DSC). DSC measurements were performed from room temperature to a maximum temperature exceeding the one observed for the highest melting component, at heating rates of 10/5/2 K/min (see Results and Discussion); as for all binary phase diagrams showing eutectic points, the onset temperatures were used to determine the *solidus* line and the eutectic temperature, while peak temperatures were used to draw the *liquidus* lines and determine the eutectic composition.^33^

### Structure Minimizations

The experimental structures (Table 2) were optimized with Materials Studio (Dmol3) using the Perdew–Burke–Ernzerhof generalized gradient approximation exchange-correlation density functional and Density functional Semicore PseudoPotentials, with atomic orbital basis set-DNP. Optimizations were considered complete when energies were converged to better than 1 × 10^–5^ Ha per atom, atomic displacements to 5 × 10^–3^ Å, and maximum forces to 2 × 10^–2^ Ha Å^–1^. The lattice parameters were kept fixed during the minimizations.

**Table 2:** Structures Used for Energy Minimization

single component	CSD Refcode	cocrystal	CCDC deposition number
Kojic acid	ZZZFMU01^34^		
3-Hydroxybenzoic acid	BIDLOP02^35^	HKA·3HBA	2220361
Imidazole	IMAZOL06,^36^ IMAZOL24^37^	HKA·Imidazole	2220359
4-Pyridone	ENISOM^38^	HKA·4-Pyridone	2220362
DABCO	TETDAM08^39^	HKA·DABCO	2220360
Urotropine	QAMCAM52^40^	(HKA)2·Urotropine	2220358

Furthermore, the experimental structures (Table 1) were optimized with CASTEP v20.11,^41^ using the Perdew–Burke–Ernzerhof generalized gradient approximation exchange-correlation density functional^42^ and ultrasoft pseudopotentials^43^ with the addition of the MBD* dispersion correction.^44^ The number of k-points were chosen to provide a maximum spacing of 2π·0.04 Å^–1^, and a basis set cutoff of 1180 eV was applied. Optimizations were considered complete when energies converged to better than 2 × 10^–5^ eV per atom, atomic displacements to 1 × 10^–3^ Å, maximum forces to 5 × 10^–2^ eV Å^–1^, and maximum stresses to 0.1 GPa. These computational settings were found to yield sufficiently accurate energies and converged minimizations. Atomic positions and lattice parameters were optimized. In the case of the DABCO cocrystal, the disorder was resolved by calculating the energy for the two potentially order structures, and for all calculations the weighted average of the two ordered structures was used.

### Cocrystal Stability

The cocrystal stability (Δ*E*_cocrystal_^stab^) was calculated based on eq 1:
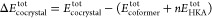
1where *E*_cocrystal_^tot^, *E*_coformer_^tot^, and *E*_HKA_^tot^ correspond to the PBE-MBD* energies of the cocrystal, coformer and HKA, respectively, and *n* to the stoichiometric ratio.

### Pairwise Intermolecular Interaction Energies

The optimized PBE-TS structures were then used as starting points for pairwise intermolecular energy calculations,^45−47^ using Crystal Explorer V17^48^ and Gaussian 16^49^ (3.8 Å radius). The intermolecular energies were calculated between all unique nearest neighbors. The model (CE-B3LYP) uses B3LYP/6-31G(d,p) molecular wave functions, calculated by applying the molecular geometries extracted from the crystal structures. This approach uses electron densities of unperturbed monomers to obtain four separate energy components: electrostatic (*E*_E_), polarization (*E*_P_), dispersion (*E*_D_), and exchange-repulsion (*E*_R_). Each energy term was scaled independently to fit a large training set of B3LYP-D2/6-31G(d,p) counterpoise-corrected energies from both organic and inorganic crystals.^47^

## Results and Discussion

### Choice of Coformers

Kojic acid is a highly soluble compound^50^ that possesses two hydrogen bond donors and four acceptors; it is also a fairly rigid molecule, with only one torsional degree of freedom shown by the hydroxymethyl group. While some metal complexes with kojic acid are known in the literature, together with their structures, neither organic cocrystals nor organic salts have been reported. A search in the Cambridge Crystallographic Database^27^ was thus conducted, via the program Mercury,^31^ to screen for suitable coformers by Molecular Complementarity^51,52^ and multicomponent Hydrogen Bond Propensity (HBP)^53,54^ methods. The HBP method was used to restrict the large number of potential coformers obtained via the molecular complementarity method. To this end, a multicomponent (MC) score was calculated: while a positive MC value indicates probable cocrystal formation, if the value is close to zero no conclusion can be formed. Tables S3 and S4 show the results of both methods, with 14 possible coformers (if scores down to zero are included). The combined use of the two methods, therefore, helps in narrowing the number of compounds that should be tried as possible coformers. It is interesting to note that in the case of piperazine and theophylline, that reacted with HKA to form stoichiometric compounds, the MC method produces “pass” flags, i.e., the prediction is of possible cocrystal formation, while the HBP method assigns to these coformers a negative value (see Tables S3 and S4). The two methods, therefore, should be tested and used as an additional tool in crystallization experiments, not as a hard-and-fast rule for molecules selection. The amino acids were also tried, but they did not give positive results, as the reagents were in the zwitterionic form, which, according to both MC and HBP searches, are not good candidates (see Tables S3 and S4).

Table 3 lists the results obtained with the coformers that were combined with HKA in various stoichiometric ratios via ball-milling, slurry, and crystallization from solution. Successful results were obtained in the case of imidazole, theophylline, pyrazine, piperazine, and 3-HBA; in all other cases physical mixtures of the starting materials were obtained, as ascertained by powder X-ray diffraction.

**Table 3:** Results of the Screening, via Ball-Milling, Crystallization, and Slurry, of Suitable Co-Formers with Kojic Acid^a^

coformer	Result	coformer	result
4-aminobenzoic acid	mixture	glutaric acid	mixture
d-pantothenol	mixture	glycine	mixture
EDTA	mixture	glycolic acid	mixture
l-arginine	mixture	**imidazole**	**cocrystal**
l-aspartic acid	mixture	maleic acid	mixture
l-glutamic acid	mixture	malonic acid	mixture
l-glutamine	mixture	nicotinamide	mixture
l-methionine	mixture	oxalic acid	mixture
l-proline	mixture	**piperazine**	**salt**
l-serine	mixture	pyrazine	mixture
l-tartaric acid	mixture	riboflavin	mixture
L-tryptophan	mixture	saccharin	mixture
l-tyrosine	mixture	sorbic acid	mixture
acetic acid	mixture	succinic acid	mixture
adipic acid	mixture	**theophylline**	**complex**^b^
benzoic acid	mixture	xanthine	mixture
citric acid	mixture	**3-hydroxybenzoic acid**	**cocrystal**
fumaric acid	mixture		

a See complete list in the Supporting Information. In bold are the positive results.

b The cocrystal/salt nature could not be established.

**Scheme 2:**
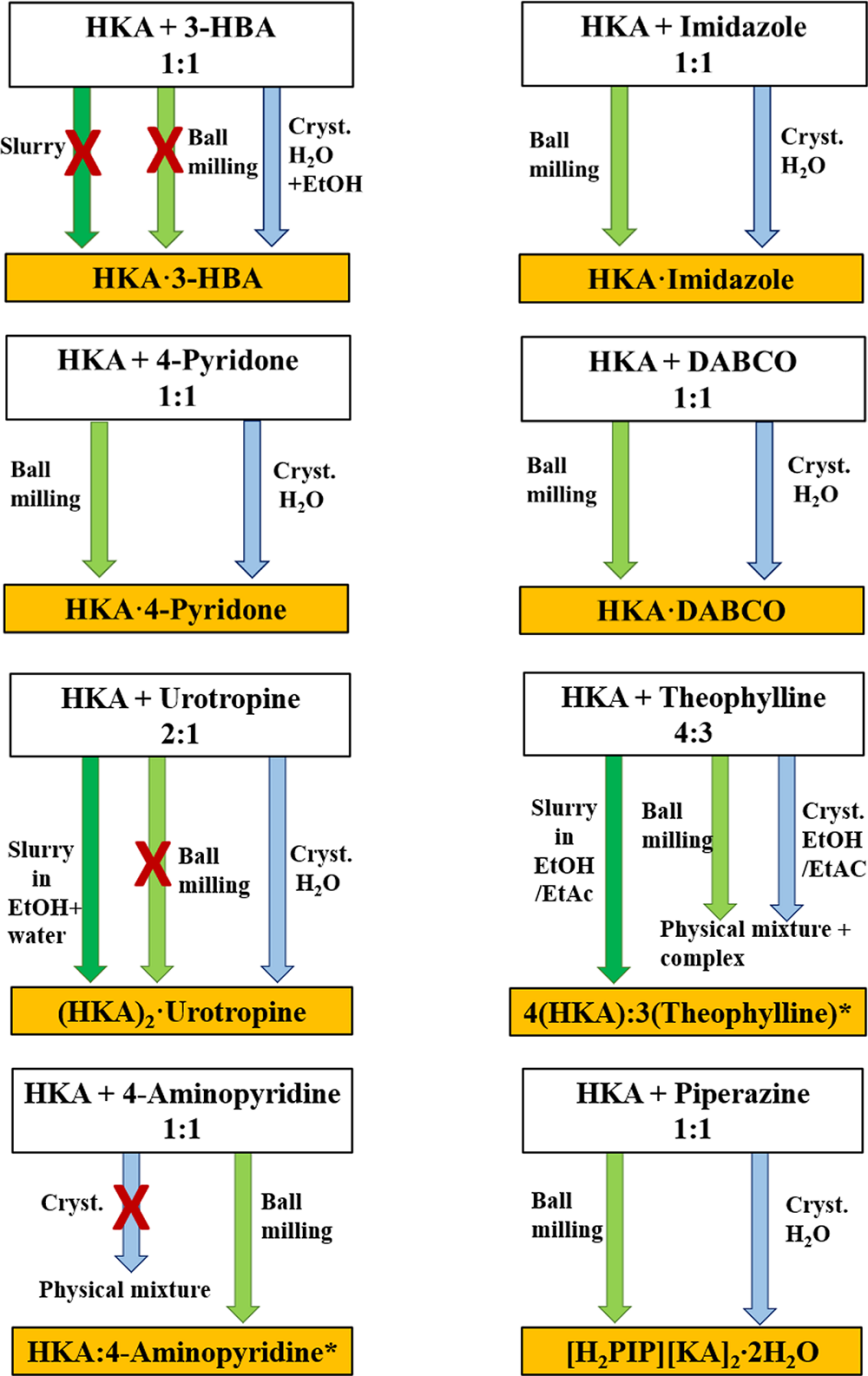
Details of the Slurry, Ball Milling, and Crystallization Processes for the Preparation of the Cocrystals and Salt Described in This Work The asterisk indicates the uncertain cocrystal/salt nature of the theophylline and 4-aminopyiridine crystalline complexes.

The reaction of HKA with the 3-hydroxybenzoic acid (3-HBA) resulted in a cocrystal with striking structural similarity with the crystal structures of both HKA and 3-HBA Form II, although the cocrystallization was conducted with 3-HBA Form I. Incidentally, the 3-HBA polymorphs have been recently investigated in detail by one of us.^55^ For this reason, we decided to further explore the possible formation of cocrystals of HKA with molecules that are known to act as coformers in cocrystals of 3-hydroxybenzoic acid. To this end, a search in the CSD was conducted, and, among other systems, we selected molecular solids possessing interesting properties in the skin care or in the antibacterial fields, and therefore they could be used as enhancers of HKA properties. A second list of possible coformers was thus compiled (see Table 4). Scheme 2 summarizes the reactions and stoichiometric ratio that resulted in the formation of the HKA cocrystals and salt described in this work.

**Table 4:** Possible, Additional Coformers of HKA Selected among Skin Care Ingredients, 3-HBA Coformers or Substances Containing Nitrogen Donor/Acceptor Groups

coformer	result	coformer	result
caffeine	mixture	**DABCO**	**cocrystal**
theobromine	mixture	**4-aminopyridine**	**complex**^b^
nicotinamide	mixture	panthenol	mixture
quinoxaline	mixture	urea	mixture
**urotropine**	**cocrystal**	salicylic	mixture
**4-pyridone**^a^	**cocrystal**		

a 4-Pyridone (IUPAC 1H-pyridin-4-one) is the predominant tautomer of 4-hydroxypyridine in liquid media and solids.^56^

b The cocrystal/salt nature could not be established.

### Solid-State Structures of HKA Cocrystals and Salt

#### HKA·3-HBA

Crystallization from solution of HKA and 3-HBA resulted in the formation of the cocrystal HKA·3-HBA. As listed in Table S2, pure 3-HBA is known to exist in three polymorphic modifications.^55^ Form I is the thermodynamically stable form, while form II and III are metastable. The commercial 3-HBA employed in the reaction with HKA was form I. Both ball milling and slurry of the solid mixture were unsuccessful, while crystallization from a water/ethanol solution resulted in the formation of HKA·3-HBA cocrystals. The cell parameters obtained from a single crystal, and listed in Table S2 together with those of HKA, are closely related to the values for 3-HBA Form II, and bear some relationship with those of crystalline HKA.

Similarly to the cocrystallization experiments, cooling crystallization of 3-HBA (from ethyl acetate) resulted in form II, whereas grinding experiments favored the formation of form I.^55^ We investigated the difference in cocrystal formation enthalpy, i.e. form I → cocrystal and form II → cocrystal. The energy difference between 3-HBA forms I and II was measured and calculated to be in the range of 0.5 to 0.6 kJ mol^–1^.^55^ Therefore, the stabilization energy (or cocrystal formation enthalpy) differs marginally, by less than 1 kJ mol^–1^_,_ if form I or II is used as the starting material.

**Figure 1:**
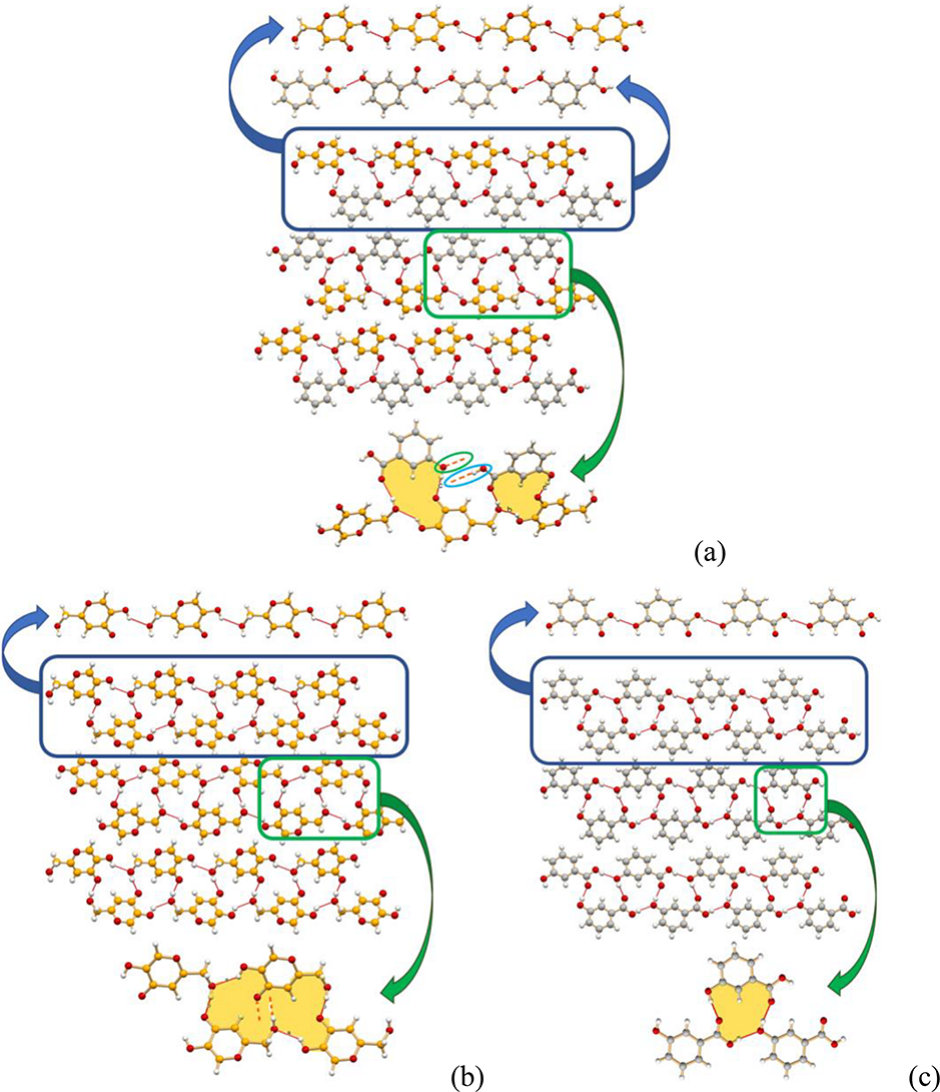
Comparison of (a) HKA·3-HBA, (b) HKA, and (c) 3-HBA form II crystal structures. Top: Hydrogen bonded chains in (a) HKA·3-HBA ring motif D_3_^3^(13) [view down the *b*-axis], (b) HKA ring motif R_4_^4^(22) [view down the *a*-axis], and (c) 3-HBA Form II motif R_3_^3^(13) ring [view down the *c-*axis]. Bottom: Detailed depiction of the seemingly identical hydrogen bonding rings; the ring motifs are partly open in (a) the cocrystal and (b) pure HKA [O_C=O_ points down, O_OH_ up, thus forming rings with molecules belonging to layers above and below the plane], while they are closed in (c) 3-HBA form II.

Figure 1a–c illustrates similarities and differences of the three packings, with special reference to the hydrogen bonding motifs. In all cases a chain motif can be detected, linking via O(H)···O_OH_ hydrogen bonds molecules of the same type (see Figure 1a). A projection in the crystallographic *ac*-, *bc*-, and *ab*-plane for the cocrystal, HKA, and 3-HBA form II, respectively, shows strong similarities in molecular arrangement, although the picture is slightly deceiving, as the hydrogen bonding rings are not equivalent, being actually “open” (hydrogen bonding to the next layer) structures in parts of the cocrystal and in HKA (see Figure 1a,b). The overall packing, however, is fairly similar, as evidenced by the cell parameters, and these hydrogen bonding similarities all concur to the possibility of replacing one every two HKA (or 3-HBA) molecules in their pure crystals, to form the stable HKA·3-HBA cocrystal. In order to obtain a deeper understanding of this behavior, we used the lattice energy calculations to investigate the cocrystal stability with respect to the parent phases (see the “Stabilization enthalpies of the co-crystals” section).

#### (HKA)_2_·Urotropine

**Figure 2:**
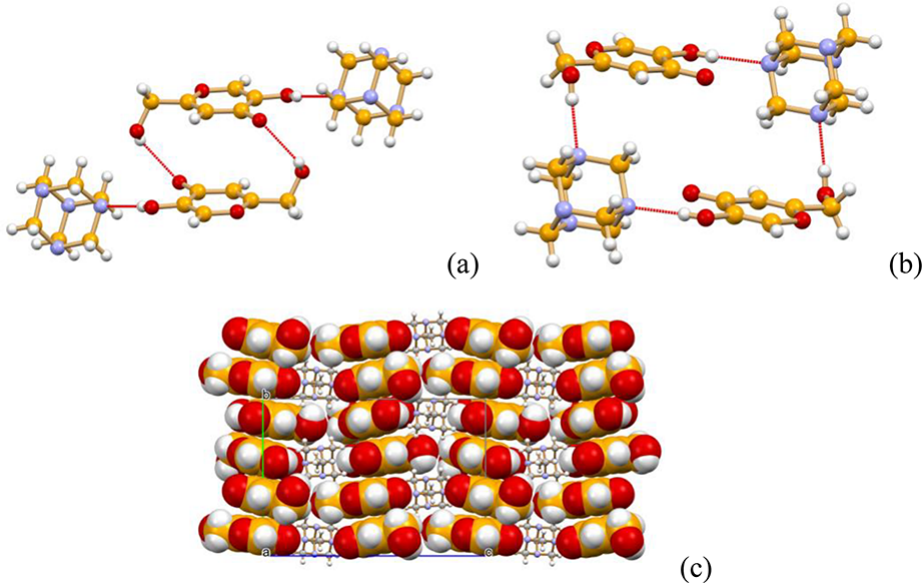
Hydrogen bonding patterns in crystalline (HKA)_2_·urotropine: (a) Hydrogen-bonded ring formed by two HKA molecules interacting via π-stacking (distance between the aromatic planes 3.48 Å) and O–H···O_CO_ bonds [O(H)···O_CO_ 2.857(7) Å], while HBs with urotropine are external [O(H)···N distances 2.764(6) Å]; (b) a large HB ring formed by alternate urotropine and HKA molecules [O(H)···N 2.692(6) and 2.793(6) Å]. (a) and (b) View down the *a*-axis. (c) A view of crystalline (HKA)_2_·urotropine down the *a*-axis, showing the stacking of HKA molecules parallel to the crystallographic *b*-axis.

Crystallization of HKA with urotropine from aqueous solution resulted in the formation of the cocrystal of formula (HKA)_2_·urotropine. Calculating powder diffraction patterns based on single crystal data matches the experimental pattern measured on the slurry product (see Figure S1). Figure 2 illustrates the main packing features and hydrogen bonding patterns. Hydrogen bonds of the O–H···O and O–H···N type between the −OH groups of HKA molecules and the nitrogen atoms of urotropine, and π-stacking between the HKA aromatic rings are the main stabilizing motifs recognizable in this cocrystal. Figure 2a shows the hydrogen-bonded ring formed by two HKA molecules interacting via π-stacking (distance between the aromatic planes 3.48 Å) and O–H···O_CO_ bonds [O(H)···O_CO_ 2.857(7) Å], while HBs with urotropine are external [O(H)···N distances 2.764(6) Å]. In Figure 2b a large HB ring is formed by alternate urotropine and HKA molecules [O(H)···N 2.692(6) and 2.793(6) Å]. The whole packing in crystalline (HKA)_2_·urotropine, shown in Figure 2c, can be seen as a stacking of HKA molecules, parallel to the crystallographic *b*-axis, with hydrogen bonds contributing as an additional “glue” to the cocrystal stability (see the Pairwise Intermolecular Energy Calculations section).

#### HKA·4-pyridone

**Scheme 3:**
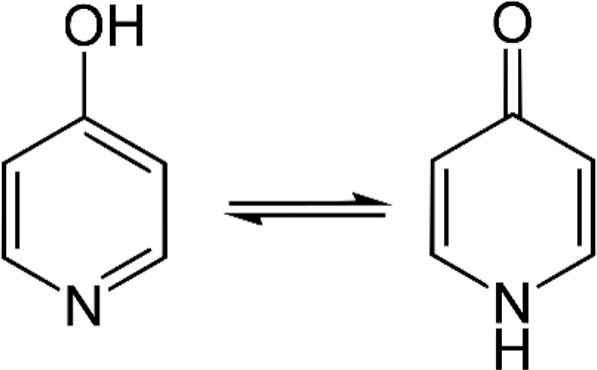
Enol- and Keto- Forms of 4-Hydroxypiridine The keto form takes the name of 4-pyridon.

The compound 4-pyridone is the predominant, keto-tautomer of 4-hydroxypyridine in liquid media and solids, as shown in Scheme 3.^56^

**Figure 3:**
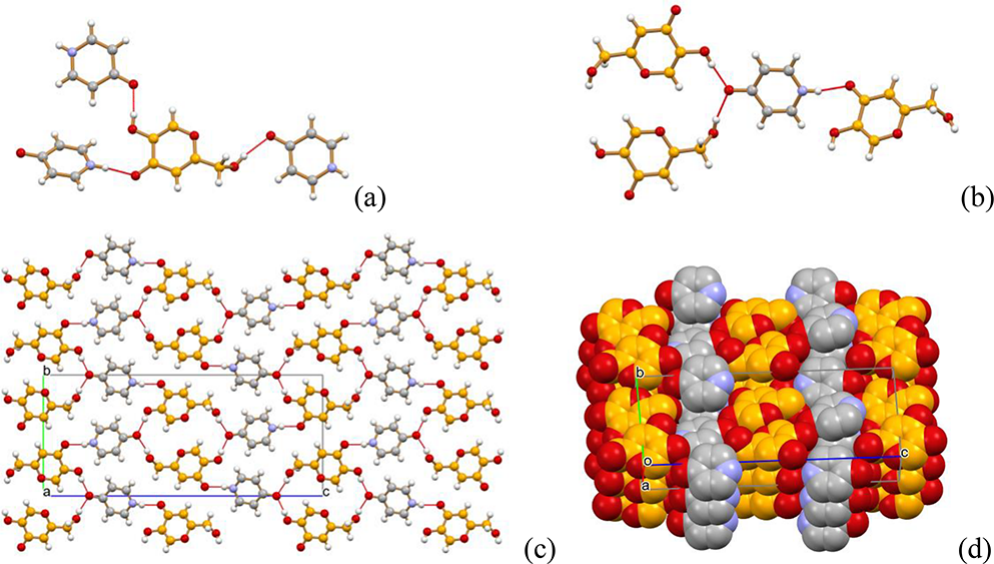
(a, b) Hydrogen-bonding interactions between HKA and the 4-pyridone molecules in crystalline HKA·4-pyridone. (c, d) Packing patterns for HKA·4-pyridone (view down the *a*-axis).

The cocrystal HKA·4-pyridone was obtained both by ball milling and by crystallization from solution of HKA and 4-pyridone (see Scheme 1). The calculated powder diffraction pattern, based on the single crystal structure, matches the experimental pattern, thus confirming that the single crystal structure is representative of the powder reaction bulk (see Figure S2). Figure 3a shows how the HKA molecule forms hydrogen bonds of the O–H···O_CO_ and O_CO_···H–N with 4-pyridone molecules only; the 4-pyridone molecule, in turn, forms hydrogen bonds of the N–H···O_CO_ and O_CO_···H–O with neighboring HKA molecules only [O···(H)O_OH_ 2.565(3) Å, O···(H)O_hydroxymethyl_ 2.752(4) Å and N(H)···O 2.717(3) Å]. A view down the crystallographic *a*-axis (see Figure 3c) shows how the hydrogen bonding requirements work together with π-stacking stabilizing interactions (see Table 8) in producing alternate layers of HKA and 4-pyridone units (Figure 3d).

#### HKA·DABCO

**Figure 4:**
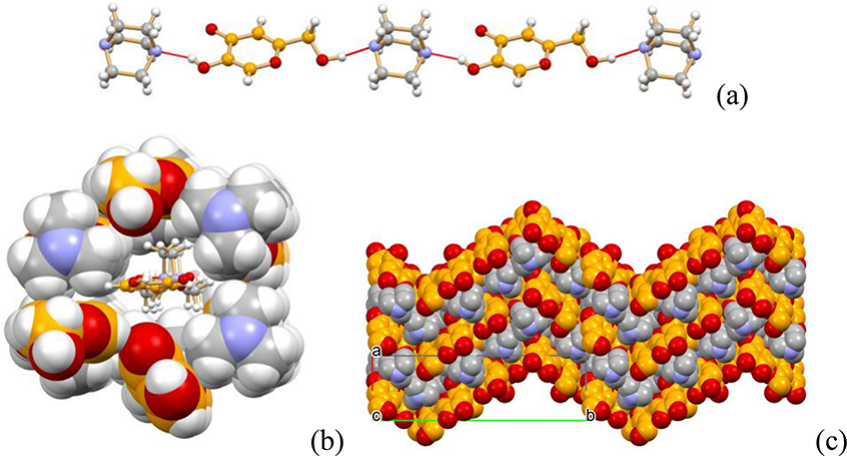
(a) Hydrogen-bonded chain involving alternating HKA and DABCO molecules and extending along the crystallographic [101] direction in crystalline HKA·DABCO; (b) each of the three independent chains present in the crystal can be seen as filling a channel formed by six neighboring, parallel chains. (c) The whole packing can also be represented as an alternation of chevron-like layers of HKA and DABCO molecules (H atoms not shown for clarity).

Manual grinding and crystallization from a solution of HKA and DABCO resulted in the 1:1 cocrystal HKA·DABCO. The identity of the bulk material produced via solution and manual grinding processes was confirmed by comparing the calculated and measured powder diffraction patterns (see Figure S3). HKA and DABCO are perfectly complementary as far as hydrogen bonding functions are involved, and the two opposite OH groups on each HKA interact with the N atoms of two neighboring DABCO molecules, thus forming an infinite chain of alternating HKA and DABCO extending parallel to the crystallographic [101] direction (see Figure 4a); along the chain the O(H)···N distances involving the hydroxyl group directly attached to the ring are shorter [N 2.615–2.636(4) Å] than the analogous distances involving the hydroxymethyl group [2.742–2.852 (5) Å]; there are three independent chains in the crystal, each formed by an independent pair of HKA and DABCO molecules, as the asymmetric unit contains three molecules of each kind. If the packing is observed along any of the three, as shown in Figure 4b, the chain looks completely embedded in a “channel” formed by six more hydrogen bonded chains. Figure 4c shows how the packing can also be seen as the convolution of two infinite, alternating chevron-like layers of HKA and DABCO molecules extending along the crystallographic *b*-axis direction.

#### HKA·Imidazole

**Figure 5:**
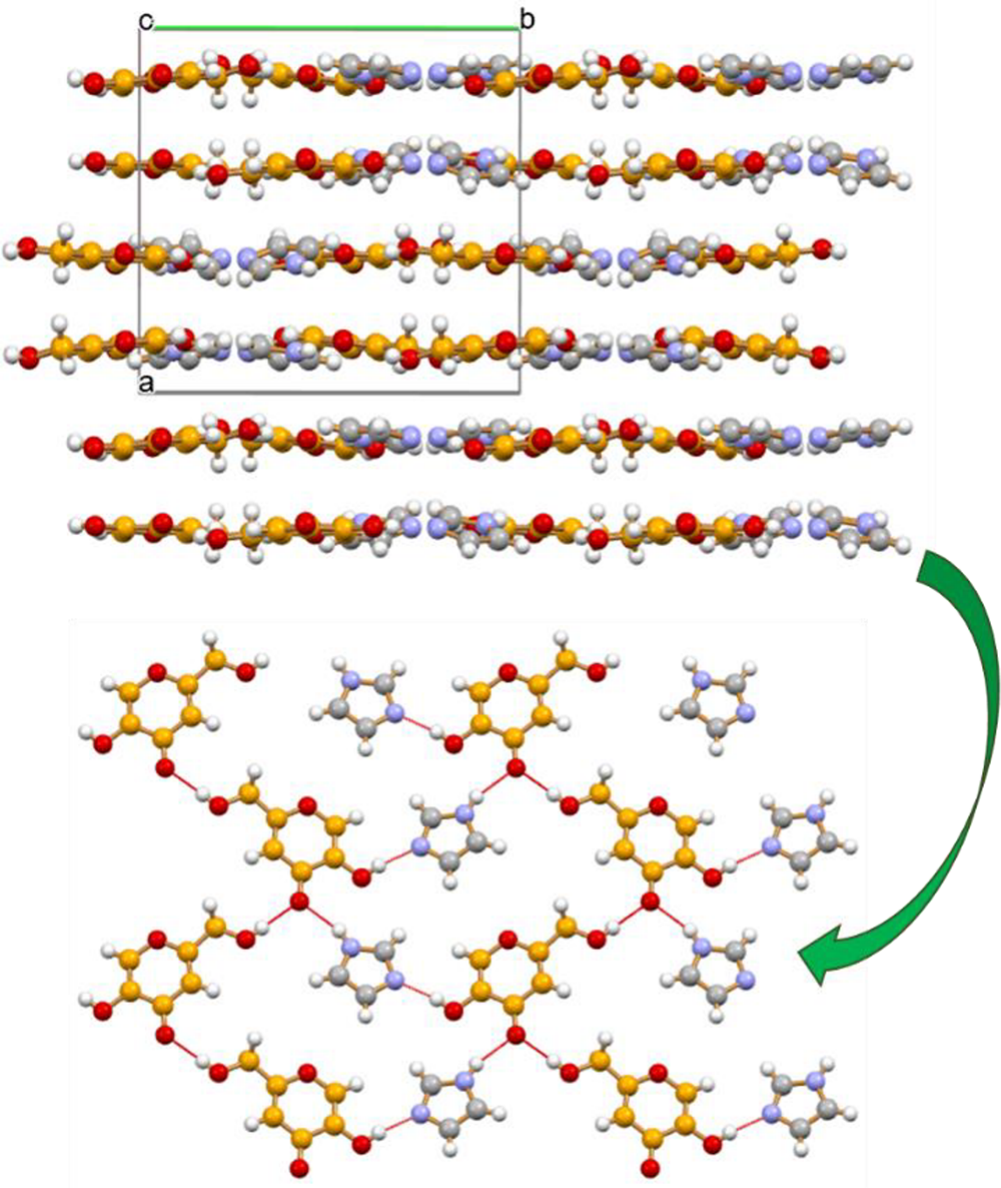
(Top) View, down the crystallographic *c*-axis, of the layered structure HKA·imidazole; (bottom) the layers are formed by hydrogen bonded chains of HKA molecules, linked via N–H···O and N···H–O hydrogen bonds by bridging imidazole molecules.

Ball milling and crystallization from a solution of HKA and imidazole yielded the 1:1 HKA·imidazole cocrystal. The measured powder diffraction patterns for the products of both processes were found superimposable to the calculated pattern (see Figure S4). The cocrystal can be described as a layered structure, as shown in Figure 5a, in which the flat layers are formed by zigzag chains of HKA molecules interacting via O–H_hydroxymethyl_···O_CO_ [O(H)···O distances 2.658(2) and 2.706(2) Å], bridged by imidazole molecules via N–H···O_CO_ [N(H)···O distances 2.795 and 2.808(3) Å] and N···H–O_hydroxyl_ [O(H)···N distances 2.612 and 2.643(2) Å).

#### (HKA)_4_·(Theophylline)_3_

Kojic acid and theophylline reacted in ball milling, slurry, and solution crystallization experiments. Unfortunately, as in the case of 4-aminopyridine, no structural solution has yet been obtained. The p*K*_a_ difference (Δp*K*_a_) between kojic acid and theophylline^57^ is ca. +0.1; therefore, according to the paper by Cruz-Cabeza on crystalline complexes and the p*K*_a_ rule,^58^ the reaction product between theophylline and kojic acid falls into the “grey zone” between cocrystals and salts, and cannot be considered with certainty a cocrystal (the probability being around 70%). In the absence of experimental evidence, we have chosen to indicate the outcome of the reaction with the term “crystalline complex”, or simply “complex”.

The assessment of the correct stoichiometry for the HKA:theophylline complex could be obtained by combined use of DSC and X-ray diffraction. Ball milling evidenced the formation of a new compound, but a clean pattern, free from reagents, could never be obtained. Slurry yielded the crystalline complex, but traces of reagents were still found (Figure S5). DSC measurements were then conducted for stoichiometric ratios yielding powder X-ray patterns with the lowest residual reagents content; as nonconsistent results were obtained, a thermal pretreatment was then conducted on the physical mixtures, that were kept overnight in an oven at 140 °C, i.e., at a temperature that does not cause HKA melting and decomposition. DSC measurements on these pretreated mixtures allowed us to observe congruent melting at 178 °C for the HKA:theophylline 4:3 stoichiometric ratio (see Figure S8). A TGA measurement showed no weight loss before decomposition; therefore, no water is present in the crystal.

We then turned to variable temperature powder X-ray diffraction, and followed the behavior with temperature, from room temperature to 175 °C, of the diffraction pattern for the 4:3 ratio. Upon heating, at a slower heating rate with respect to the DSC experiments, the complex is indeed formed starting at ca. 135 °C; as the reaction involving the mixture is slow, and unreacted kojic acid is still present at its decomposition temperature (around 155 °C), theophylline peaks are still detectable, and become more evident at 175 °C, close to the melting point of the complex (see Figure S6). The same thermal pretreatment procedure utilized for DSC measurements was then applied to the 4:3 mixture, resulting in a pure complex, as shown in Figure S7, thus confirming the stoichiometric ratio as HKA:theophylline = 4:3.

#### HKA·4-Aminopyridine

The cocrystal HKA·4-aminopyridine was obtained by ball milling of a 1:1 stoichiometric ratio for the two reagents. The X-ray powder pattern of the resulting product (see Figure S10) does not contain peaks of the reagents; a DSC trace (Figure S11) shows that melting of the cocrystal occurs at 58 °C, i.e., at very low temperature, as both HKA and 4-aminopyridine melt at 155 °C. The TGA trace of Figure S12 shows that decomposition of the liquid phase starts around 100 °C. Unfortunately, no single crystals have yet been obtained, nor a powder diffraction pattern of sufficient quality for a structural solution from powder to be obtained. As in the case of theophylline, the Δp*K*_a_ between 4-aminopyridine and kojic acid, equal to +1.5,^59^ places the product of the reaction within the “grey zone” between cocrystals and salts, with an almost equal probability of being one or the other form; we have thus indicated the outcome of the reaction a “crystalline complex”.

#### [H_2_PIP][KA]_2_·2H_2_O

**Figure 6:**
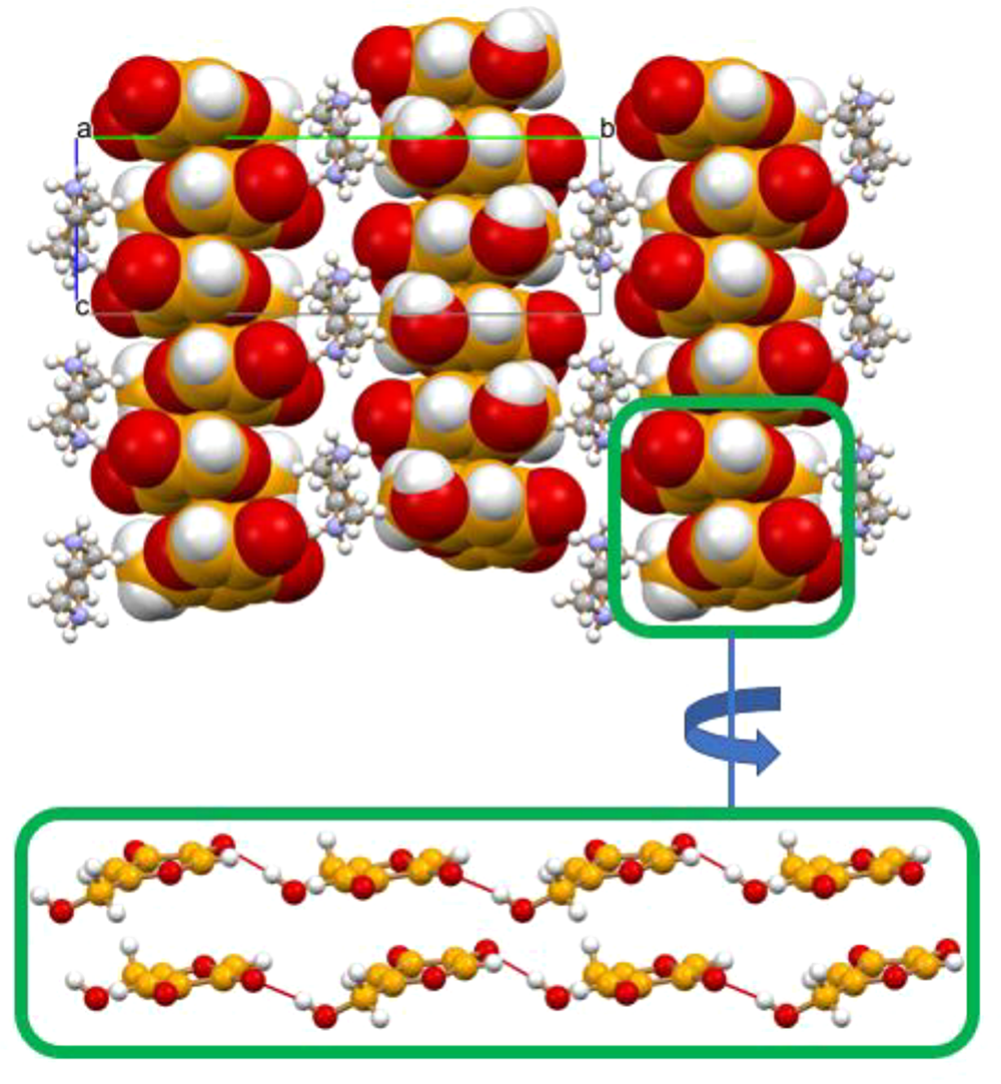
Layers of π-stacked hydrogen bonded kojiate chains in crystalline [H_2_PIP][KA]_2_·2H_2_O. The piperazinium cations and the water molecules are distributed between the anionic layers.

The dihydrate molecular salt [H_2_PIP][KA]_2_·2H_2_O was obtained by ball milling and solution crystallization (see Scheme 2). The calculated and measured powder diffraction patterns, shown in Figure S13, confirm that the single crystal structure is representative of the bulk products. Figure 6 shows the packing arrangement of the piperazinium and kojate ions in the crystal: it can be seen that the aromatic plane of the kojate anions is π-stacked along the crystallographic *c*-axis, at a distance of ca. 3.4 Å, forming layers of columns intercalated by the piperazinium cations.

**Figure 7:**
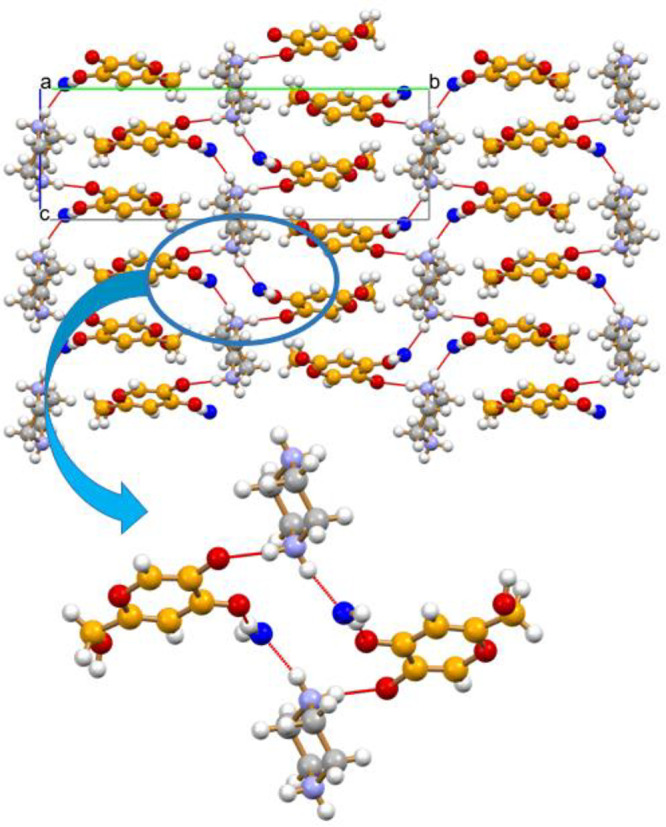
Hydrogen-bonding interactions connecting the kojiate anions, the piperazinium cations and the water molecules, and an enlargement of the large hydrogen bonded rings parallel to the *bc*-plane. Each kojiate anion is hydrogen bonded to anions below and above the plane, as previously shown in Figure 6.

**Figure 8:**
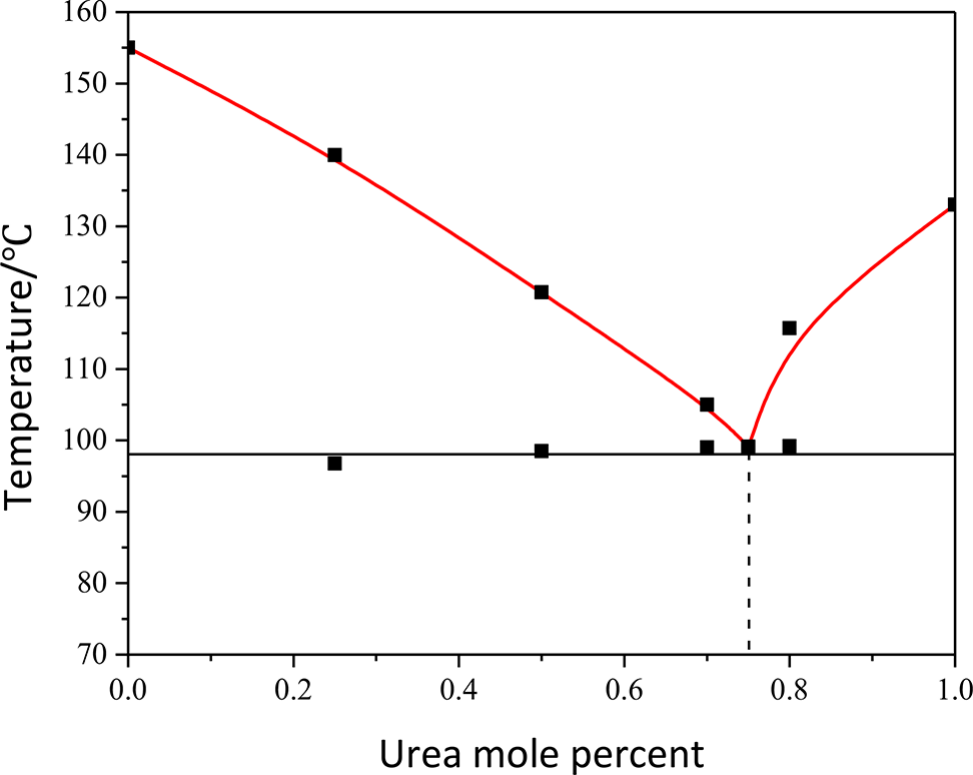
Binary phase diagram of the HKA:urea system, showing a eutectic point at 0.75 mole fraction of urea.

Figure 7 also shows how the columns are connected within each layers via O–H_hydroxymethyl_···^(−)^O hydrogen bonds [O(H)···^(−)^O distance 2.66(2) Å]. The stacking of anions is made possible by the highly stabilizing electrostatic contribution of the interlayers cation···anion interactions (see Figure S35), with the addition of charge assisted hydrogen bonds of the N–H···^(−)^O type and bridging water molecules, as shown in Figure 8 [N(H)···^(−)^O 2.747(2) Å, N(H)···O_CO_ 2.882 (2) Å, N(H)···O_W_ 2.715(2) Å, O(H)_W_···O_CO_ 2.685(2) Å, and O(H)_W_···^(−)^O 2.710(2) Å].

### Binary Phase Diagrams and Eutectic Systems

Cocrystal formation could not be observed for the remaining selected coformers, despite the many attempts with different cocrystallization techniques (solution, slurry, or mechanochemical methods), stoichiometric ratios, solvents, and temperatures. In the end, we decided to investigate the phase diagrams of a small number of binary systems, to determine their eutectic composition and temperature: eutectic compositions are currently recognized as a possible alternative, when cocrystal formation is not possible, to modify the physical properties of a compound, such as thermal stability and solubility.^60−64^ We thus prepared via ball-milling (see Experimental Section) solid-state mixtures of HKA containing mole fractions from 0 to 1 of the second component of interest. DSC measurements were then performed, and the resulting thermograms (see Figures S14–S18) used for the construction of the binary phase diagrams (see Experimental Section). Table 5 lists the eutectic compositions and temperatures for the systems analyzed. The eutectic mixtures were also analyzed via powder X-ray diffraction (see Figures S19–S22). In all cases the patterns confirm the presence of physical mixtures, as no peaks are observed in addition to the peaks of the two components.

**Table 5:** Eutectic Compositions and Temperatures for the Four Eutectic Systems

eutectic composition	eutectic temperature (°C)	mp second component^a^ (**°**C)
HKA:urea 1:3	98	135
HKA:salicylic acid 1:1	119	160
HKA:panthenol 1:3	62	67
HKA:nicotinamide 2:3	97	130

a mp of HKA is 155 °C.

As it can be observed in Figure 8, the phase diagram of the HKA:urea system illustrates the formation of a eutectic at a temperature of ca. 98 °C and a urea mole fraction of 0.75 (this corresponds to a 1:3 stoichiometric ratio).

**Figure 9:**
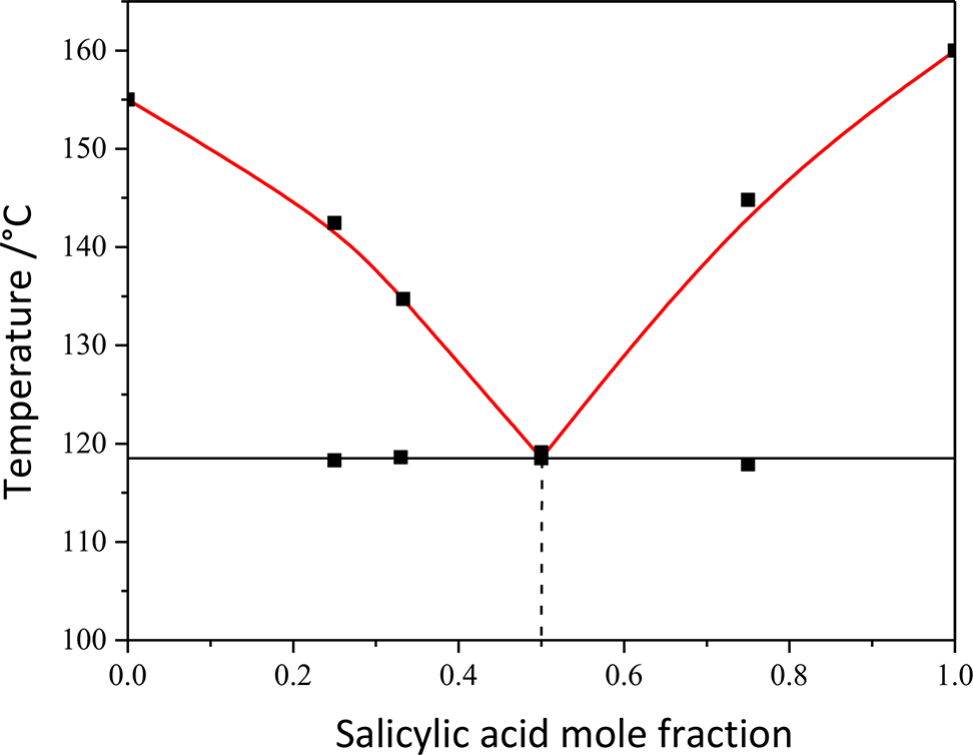
Binary phase diagram of the HKA:salicylic acid system, showing a eutectic point at 0.5 mole fraction of salicylic acid.

Figure 9 shows the phase diagram of the HKA:salicylic acid system. A eutectic formed at a temperature of ca. 119 °C, for a 1:1 stoichiometric ratio of HKA and salicylic acid. In both cases, the eutectic composition is characterized by a large depression of the melting point with respect to the pure components.

**Figure 10:**
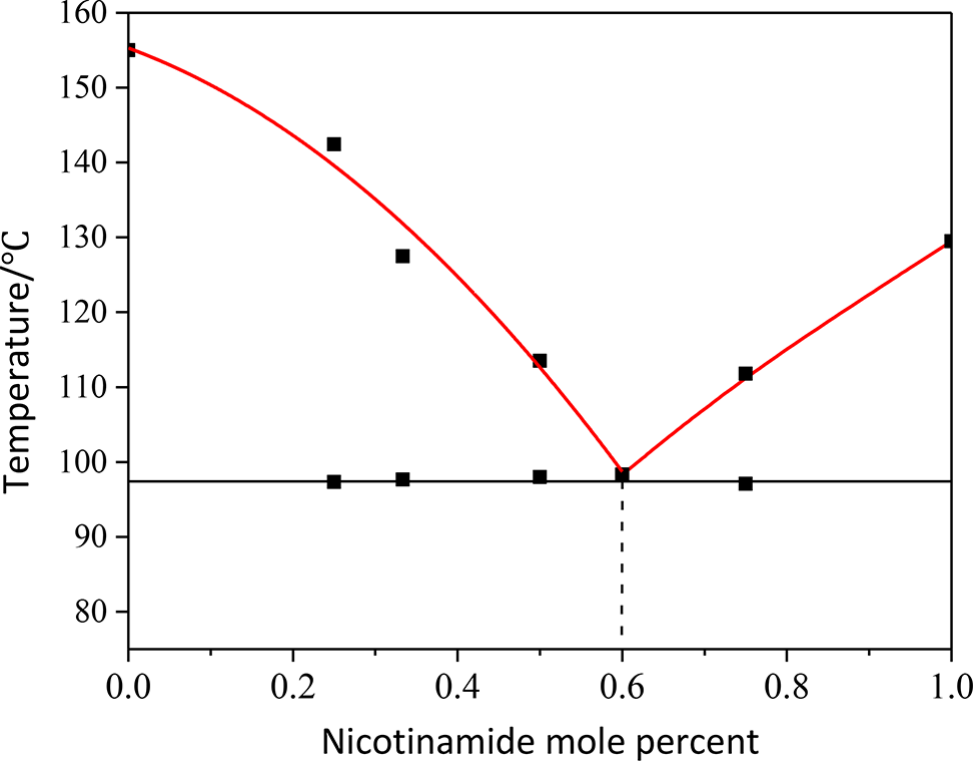
Binary phase diagram of the HKA:nicotinamide system, showing a eutectic point at 0.6 mole fraction of nicotinamide.

The phase diagram of the HKA:nicotinamide binary system is shown in Figure 10. As in the previous two cases, the eutectic point for this system is found at a temperature, 97 °C, that is much lower than the melting point of both components; the eutectic composition corresponds to a 0.6 molar fraction of nicotinamide, i.e., to a 2:3 stoichiometric ratio.

**Figure 11:**
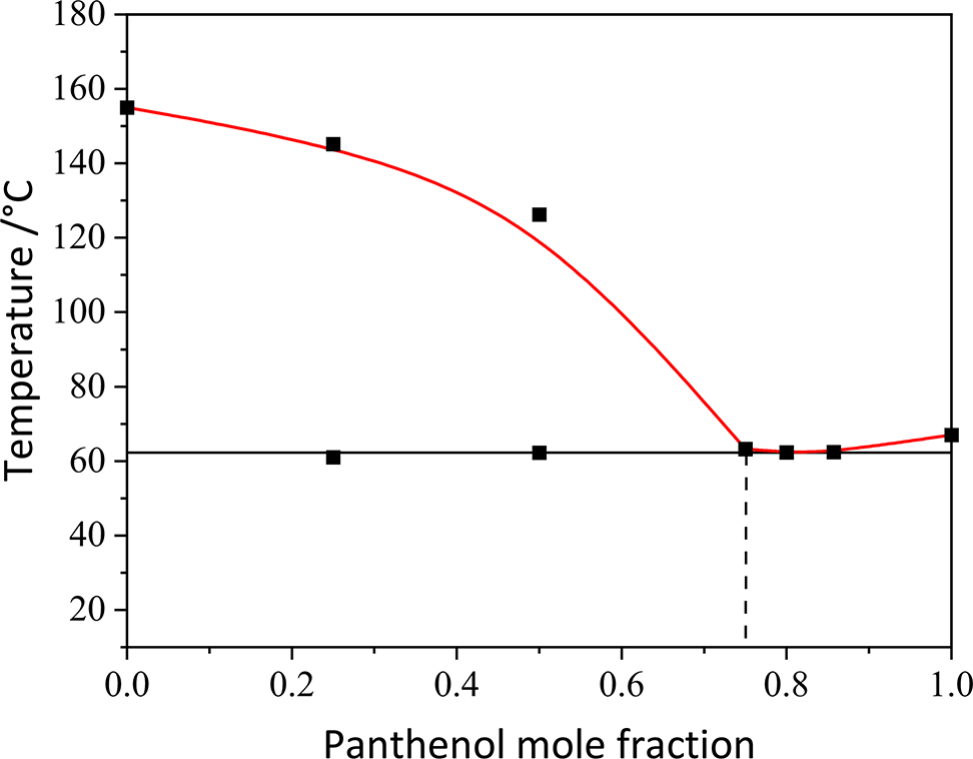
Binary phase diagram of the HKA:panthenol system, showing a eutectic point at 0.75 mole fraction of panthenol.

The HKA:panthenol phase diagram is shown in Figure 11. A eutectic point can be seen at ca. 62 °C, corresponding to a mole ratio of HKA to panthenol of 1:3. In the case of the HKA:panthenol binary system, the effect of the addition of panthenol to kojic acid is pronounced only for high molar fraction of panthenol, as can be seen from the composition of the eutectic mixture, corresponding to a 0.75 molar fraction of panthenol. The eutectic temperature is very close to the melting point of panthenol, to the point that the DSC thermogram (see Figure S18) show only a widening of the peaks due to melting, and no separation of the solidus/liquidus temperature is possible for a molar fraction of panthenol higher than 0.75.

### Periodic Electronic Structure Calculations

The chosen optimization method was successful in reproducing the experimental structures (Table 6). The structures were compared using the Molecular Similarity Module in Mercury to determine the root-mean-square deviation of the non-hydrogen atoms in a cluster of 15 molecules (rmsd_15_).^65^

**Table 6:** Comparison of the Experimental Structures with Their PBE-MBD* Optimized Counterparts

		unit cell parameters						
crystal structure	space group	*a* (Å)	*b* (Å)	*c* (Å)	β (deg)	*E*^tot^/kJ mol^–1^	cell volume (Å^3^)	rmsd_15_ (Å)
3HBA	*Pna*2_1_	20.085(2)	3.7591	8.2934(10)	90		626.164	
PBE-MBD*	*Pna*2_1_	19.5325	3.7063	8.2186	90	–243086.11	594.972	0.12
HKA	*P*2_1_/*n*	3.8323(8)	18.409(6)	8.505(4)	96.56		596.089	
PBE-MBD*	*P*2_1_/*n*	3.7629	18.1069	8.3985	96.838	–270121.99	568.157	0.07
cocrystal	*P*2_1_/*c*	20.2173(9)	3.8191(3)	16.3559(7)	101.105(4)		1239.22	
PBE-MBD*	*P*2_1_/*c*	19.7370	3.7965	16.1507	101.796	–513209.73	1184.64	0.12
urotropine	*I*-43*m*	6.56360(10)	6.56360(10)	6.56360(10)	90		282.765	
PBE-MBD*	*I*-43*m*	6.7999	6.7999	6.7999	90	–216675.0447	314.418	0.21
cocrystal	*P*2_1_/*c*	6.9434(4)	14.0701(10)	20.0540(15)	96.395(6)		1946.97	
PBE-MBD*	*P*2_1_/*c*	6.7226	13.9418	19.5708	97.704	–756920.1533	1817.68	0.25
imidazole_al^a^	*P*2_1_/*c*	7.569(1)	5.366(1)	9.785(2)	119.08(1)		347.322	
PBE-MBD*	*P*2_1_/*c*	7.516	5.3513	8.9737	108.628	–105272.1941	342.018	0.04
imidazole_be^a^	*Aba*2	13.875(15)	8.675(13)	5.301(14)	90		638.058	
PBE-MBD*	*Aba*2	14.4347	8.5353	5.2379	90	–105269.4355	645.333	0.12
cocrystal	*P*2_1_/*c*	13.8505(8)	13.7616(4)	11.0176(5)	108.155(5)		1995.47	
PBE-MBD*	*P*2_1_/*c*	13.5105	13.6946	10.8664	109.335	–375398.7053	1897.12	0.13
pyridone	*C*2/*c*	9.5932(8)	9.4565(6)	10.7738(10)	114.687(11)		888.049	
PBE-MBD*	*C*2/*c*	9.5557	9.4038	10.3869	114.912	–152970.084	846.521	0.14
cocrystal	*P*2_1_/*c*	3.9189(5)	10.8816(11)	25.187(2)	90.888(9)		1073.94	
PBE-MBD*	*P*2_1_/*c*	3.8591	10.7775	25.0851	90.228	–423104.4374	1043.32	0.08
DABCO	*P*6_3_/*m*	6.0798(4)	6.0798(4)	9.3167(13)	90		298.244	
PBE-MBD*	*P*6_3_/*m*	5.9765	5.9765	9.2479	90	–163342.3208	286.067	0.09
cocrystal	*P*2_1_/*c*	10.1484(5)	32.2238(14)	12.0448(6)	106.208(5)		3782.34	
PBE-MBD*^b^	*P*2_1_/*c*	10.0922	31.4866	11.9163	105.888	–433468.0326	3641.98	0.11
PBE-MBD*^b^	*P*21/*c*	9.9862	31.7424	11.9174	105.211	–433468.0126	3645.30	0.09

a Calculations have been carried out for the alpha (al) and beta (be) polymorph.

b The experimental structure shows a 50:50 disorder of the −CH_2_–OH group. Values for the two ordered structures are given.

In the case of [H2PIP][KA]_2_·2H_2_O, the salt and a hypothetical cocrystal (proton moved) were used as input for structure optimization calculations (PBE-MBD*). Proton transfer to the salt was observed for the hypothetical cocrystal structure. The experimental salt structure was obtained in both cases after structure optimization. Thus, the calculations reflect the experimental finding, i.e. salt instead of cocrystal formation.

### Stabilization Enthalpy of the Cocrystals

The stabilization enthalpy was calculated for each of the cocrystal according to eq 1, and the results are given in Table 7. Δ*E*_cocrystal_^stab^ is negative for an exothermic reaction, indicating that the cocrystal should be stable with respect to its components (ignoring entropic effects). The enthalpy was estimated to be negative for the five cocrystals, indicating that for all cocrystals are enthalpically stabilized. The comparison of the Dmol3 calculations (no cell optimization) to the PBE-MBD* optimizations reveals that the cheaper (with respect to computational time) method already indicates the trend, i.e., cocrystal formation, albeit some of the Δ*E*_cocrystal_^stab^ values differ between the two applied methods.

**Table 7:** Stabilization Enthalpies of the HKA Cocrystals

Δ*E*_cocrystal_^stab^/ kJ mol^–1^	PBE-MBD*	Dmol3
3-HBA	–1.63	–1.57
Urotropine	–1.13	–10.26
Imidazole	–4.52	–6.26
4-Pyridone	–12.36	–18.85
DABCO	–3.71	–2.0

### Pairwise Intermolecular Energy Calculations

The strongest pairwise interactions (hydrogen-bonding and aromatic) observed in the HKA, coformer, and cocrystal structures are given in Table 8, and the full list is provided in the Supporting Information (Table S6).

**Table 8:** Strongest Pairwise Intermolecular Interactions^a^ Seen in HKA, Coformers (3-HBA, Imidazole, and 4-Pyridone), and Cocrystals

			kJ mol^–1^				
compound	interaction type	*n*	*E*_E_/kJ	*E*_P_	*E*_D_	*E*_R_	*E*_tot_^b^
HKA	O–H···O	2	–73.8	–18.8	–12.5	94	–44.8
	O–H···O	2	–43.2	–7.5	–10.8	57	–25.4
	π···π	2	–7.6	–2.9	–36.5	32.7	–21.8
3-HBA	O–H···O	2	–70	–16.1	–12	90.1	–40.7
	O–H···O	2	–57.5	–11.7	–10.6	76.9	–31.1
	π···π	2	–2.6	–1.4	–36	24.4	–20
cocrystal	O–H···O	2	–78.3	–18.9	–10.8	97.9	–45.8
	O–H···O	2	–61.4	–15.2	–13.9	76	–41.4
	O–H···O	2	–47.1	–7.8	–9.6	57.8	–28.2
	O–H···O	2	–56.8	–12.1	–10.3	81.3	–27.8
	π···π	2	–7.1	–2.9	–36.2	31.1	–21.9
	π···π	2	–0.1	–1	–31.7	17.8	–17.4
imidazole	N–H···O	2	–69.3	–17.2	–8.6	79.2	–44.6
	π···π	1	–10.9	–1.7	–12.4	5.5	–20.2
	C–H···π	2	–8.5	–1.3	–13.1	9.3	–15.7
cocrystal (*Z*′ = 2)	O–H···N	2	–101.2	–25.9	–12.1	135.1	–53.2
	N–H···O	2	–63.6	–16.1	–12.1	63.5	–50.5
	N–H···O	2	–65.7	–16.4	–12.4	68.8	–49.9
	O–H···N	2	–105.6	–26.9	–12.7	150.7	–49.5
	O–H···O	2	–73.5	–18.3	–11.7	95.1	–42.7
	O–H···O	2	–68.8	–17	–12.1	87.5	–41.7
4-pyridone	N–H···O	2	–71.6	–17.2	–6.5	67.6	–52.3
	π···π	1	–24.5	–3.5	–27.1	20.8	–39.2
	π···π	1	–23.5	–3.5	–23.4	14.9	–38.6
cocrystal	O–H···O	2	–109.2	–28.8	–11.7	121.9	–71.6
	N–H···O	2	–82.5	–20.1	–13.4	92.6	–56.6
	O–H···O	2	–78.8	–21	–11.8	85.6	–56.3

a Times the interaction is present (*n*), electrostatic (*E*_E_), polarization (*E*_P_), dispersion (*E*_D_), and exchange-repulsion (*E*_R_).

b *E*_tot_ = *k*_E_*E*_E_ + *k*_P_*E*_P_ + *k*_D_*E*_D_ + *k*_R_*E*_R_, with *k* being scale factors.

Based on the coformers the five cocrystals were divided into two groups: (1) coformers with hydrogen-bonding donor and hydrogen-bonding acceptor capabilities and (2) coformers featuring only hydrogen-bonding acceptor groups. A comparison of the intermolecular interactions seen in HKA, 3-HBA, imidazole, pyridine, and their respective cocrystal reveals that the strongest interaction is always formed in the cocrystal, despite the fact that HKA and coformers can form strong interactions with the single component states (Table 8). Furthermore, the strongest interaction is exclusively formed between HKA and the coformer. Overall, the electrostatic (hydrogen-bonding) and dispersion forces (aromatic interactions) significantly contribute to the stability of the crystal lattice of the cocrystals, coformers, and HKA. It is also noteworthy that for the *Z*′ = 2 cocrystal (imidazole HKA) the two independent molecules form interactions of similar strengths. The strongest interaction, −71.6 kJ/mol (Table 8), is seen between HKA and 4-pyridone, and this interaction, being present twice, may rationalize the relatively high Δ*E*_cocrystal_^stab^ value calculated for this cocrystal.

Both, DABCO and urotropine, do not exhibit strong hydrogen-bonding donor groups. Therefore, it is not surprising that the pairwise interactions seen in the cocrystal are of higher strengths than in the coformer alone (Table 9). The cocrystal interactions are even stronger than the strongest interaction seen in HKA. In the case of the DABCO cocrystal very strong electrostatic contributions and strong dispersion forces contribute to its stability. The two ordered structures differ slightly in the strengths of the intermolecular interactions, but differ by less than 0.1% in the sum of all intermolecular interactions. Thus, the slightly weaker O–H···N interaction in orientation 1 (Table 9) is compensated for by the other intermolecular interactions. The urotropine cocrystal is the only cocrystal in which the strongest pairwise interaction is formed between two HKA molecules and the only 2:1 cocrystal. Thus, the interaction features of the HKA molecule are present twice. Furthermore, this cocrystal exhibits a very strong pairwise intermolecular interaction (−72.4 kJ/mol), which is present only once as opposed to the nearly equi-energetical interaction seen in the 4-pyridone cocrystal.

**Table 9:** Strongest Pairwise Intermolecular Interactions^a^ Seen in HKA, Coformers (DABCO and Urotropine), and Cocrystals

			kJ mol^–1^				
compound	interaction type	*n*	*E*_E_/kJ	*E*_P_	*E*_D_	*E*_R_	*E*_tot_^b^
HKA	O–H···O	2	–73.8	–18.8	–12.5	94	–44.8
	O–H···O	2	–43.2	–7.5	–10.8	57	–25.4
	π···π	2	–7.6	–2.9	–36.5	32.7	–21.8
DABCO	O–H···N	2	–77.1	–17.9	–19.5	101.4	–49.1
cocrystal (*Z*′ = 3)	O–H···N	2	–69.5	–18.5	–17	85.7	–49
orientation 1	O–H···N	2	–54.8	–15.3	–17.2	57.5	–48.7
	O–H···N	2	–69.9	–16	–19.1	90.8	–46.3
	O–H···N	2	–69.7	–15.9	–19.3	92	–45.5
	O–H···N	2	–29.4	–10.5	–15.8	40.2	–27.8
DABCO	O–H···N	2	–89.6	–21.8	–18.7	126.5	–48.9
cocrystal (*Z*′ = 3)	O–H···N	2	–73.3	–17.9	–17.4	95.1	–47.2
orientation 2	O–H···N	2	–74.7	–18.1	–17.7	99.4	–46.5
	O–H···N	2	–57.3	–16.1	–16.0	65.8	–45.7
	O–H···N	2	–66.2	–17.6	–15.3	83.9	–44.6
	O–H···N	2	–71.0	–18.5	–15.1	95.4	–42.9
urotropine	C–H···N	8	–9.8	–2.6	–21.2	17.4	–19.9
cocrystal	O–H···O/π···π	1	–97.5	–22	–34.9	125.2	–72.4
	O–H···N	2	–78.8	–22	–25.6	107.3	–55.6
	O–H···N	2	–72.3	–16.8	–21.3	86	–54.3
	O–H···N	1	–78.2	–18.7	–19	97.2	–53.1

a Times the interaction is present (*n*), electrostatic (*E*_E_), polarization (*E*_P_), dispersion (*E*_D_), and exchange-repulsion (*E*_R_).

b *E*_tot_ = *k*_E_*E*_E_ + *k*_P_*E*_P_ + *k*_D_*E*_D_ + *k*_R_*E*_R_, with *k* being scale factors.

## Conclusions

In this paper we have reported our experimental and computational results obtained in the investigation of a family of cocrystals of kojic acid. The large number of unsuccessful experiments has confirmed the poor cocrystallization tendency of this important organic compound. Nonetheless, we have been able to identify and structurally characterize five new cocrystals with the cocoformers 3-hydroxybenzoic acid (3-HBA), imidazole, 4-pyridone, DABCO, and urotropine; in the case of the stoichiometric compounds of kojic acid with theophylline and 4-aminopyridine no full structural characterization was achieved, and therefore the distinction between salt and cocrystal with could not be made with certainty. In the case of panthenol, nicotinamide, urea, and salicylic acid their mixtures with kojic acid were investigated via differential scanning calorimetry and powder diffraction, and their eutectic compositions and temperatures determined. In contrast, proton transfer and salt formation have been observed in the case of piperazine.

The stability and intermolecular interactions in all structurally characterized compounds have been unraveled based on periodic electronic structure calculations and pairwise interaction energy estimations. Furthermore, the calculations for [H_2_PIP][KA]_2_·2H_2_O are in agreement with the experimental findings, the formation of a molecular salt instead of a cocrystal.

As pointed out in the introduction, the main motivation of this work stems from the need to contribute to the growing challenge posed by the increase in antimicrobial resistance by pathogens, due to the overuse of antibiotics. For example, some of us have reported that the antibiotic activity of the well-known antibiotic ciprofloxacin can be strengthened via cocrystallization with the natural products carvacrol and thymol.^66^ Analogously, we have shown that the antimicrobial compound proflavine, and recently also kojic acid, can be cocrystallized with metal salts of silver, zinc, and gallium to increase the minimal inhibition concentration of the drug activity toward bacteria.^67−69^ The preparation of cocrystals of known drugs, as well as the quest for new combinations of natural antibacterials, have been shown to be successful in the development of improved or novel drugs; extensive work of other research groups is well documented in a recent review by Nangia.^70^

As a logical follow-up of the results reported in this study, we are now investigating the antimicrobial and bacteriostatic behavior of the novel cocrystals discussed above. The results will be reported in the near future in specialized publications.

## Supporting Information Available

Single-crystal and powder X-ray diffraction, DSC and TGA measurements, virtual cocrystal screening, pairwise intermolecular energy calculations (PDF)
