# Supplementary material for: Searching for Suitable Kojic Acid Coformers: From Cocrystals and Salt to Eutectics
Source: Cryst Growth Des. 2023 Feb 9;23(3):1874–87. doi: 10.1021/acs.cgd.2c01364 (PMC9983005; doi:10.1021/acs.cgd.2c01364)
Supplement: Supplementary file 1 — cg2c01364_si_001.pdf [file cg2c01364_si_001.pdf]

Supporting Information

(32 pages)

Searching for suitable kojic acid co-formers: from co-crystals and salt to eutectics.

Renren Sun, Doris E. Braun, Lucia Casali, Dario Braga, Fabrizia Grepioni

Single Crystal X-ray Diffraction………………………………………………………………………..……. 2

Powder X-ray diffraction, DSC and TGA……………………………………………………………….... 3

Co-former search (CSD)..14

Co-crystallization experiments..19

Pairwise intermolecular interactions.……………………………………………………………..……… 21

Energy framework diagrams………………………………………………………………………………… 26

References…… 32

Single Crystal X-ray Diffraction

Table S1. Single crystal data and details of measurements for the HKA·3-HBA, HKA·Imidazole, HKA·4-Pyridone, HKA·DABCO, 2HKA·Urotropine co-crystals and the $[H_2PIP][KA]_2 \cdot 2H_2O$ salt.

**Compound**	**HKA·3-HBA**	**HKA·Imidazole**	**HKA·4-Pyridone**	**HKA·DABCO**	**2HKA·Urotropine**	**$[H_2PIP][KA]_2 \cdot 2H_2O$**
Chemical formula	$C_{13} H_{12} O_7$	$C_9H_{10}N_2O_4$	$C_{11}H_{11}NO_5$	$C_{36}H_{54}N_6O_{12}$	$C_{18}H_{24}N_4O_8$	$C_{16}H_{26}N_2O_{10}$
Formula weight	280.23	210.19	237.21	762.85	424.41	406.38
Morphology, color	Needle, colorless	Block, brown	Block, colorless	Plate, light yellow	Block, brown	Plate, colorless
Crystal system	Monoclinic	Monoclinic	Monoclinic	Monoclinic	Monoclinic	Monoclinic
Space group	$P2_1/c$	$P2_1/c$	$P2_1/c$	$P2_1/c$	$P2_1/c$	$P2_1/c$
a (Å)	20.2173(9)	13.8505(8)	3.9189(5)	10.1484(5)	6.9434(4)	6.8950(7)
b (Å)	3.8191(3)	13.7616(4)	10.8816(11)	32.2238(14)	14.0701(10)	20.0467(7)
c (Å)	16.3559(7)	11.0176(5)	25.187(2)	12.0448(6)	20.0540(15)	6.9058(3)
α (°)	90	90	90	90	90	90
β (°)	101.105(4)	108.155(5)	90.888(9)	106.208(5)	96.395(6)	101.917(6)
γ (°)	90	90	90	90	90	90
V (Å^3)	1239.22(12)	1995.46(17)	1073.9(2)	3782.3(3)	1947.0(2)	933.96(11)
Z	4	8	4	4	4	2
F(000)	584	880	496	1632	896	868
ρ_{calc}(g·cm^{-3})	1.502	1.399	1.467	1.340	1.448	1.445
μ(mm^{-1})	0.124	0.112	0.117	0.101	0.115	0.121
N_{reflns}, N_{par}	2806, 185	4583, 275	2426, 156	8735, 493	4172, 275	1583, 134
R_1 [on F_0^2, $I>2\sigma(I)$]	0.0835	0.0692	0.0831	0.0992	0.1042	0.0343
wR_2(all data)	0.1835	0.1617	0.1458	0.2486	0.3512	0.0868

Table S2. Crystal data comparison for trimorphic 3-HBA,[1] kojic acid and the co-crystal HKA·3-HBA.

**Compound**	**3-HBA form I (BIDLOP)**	**3-HBA form II (BIDLOP02)**	**3-HBA form III[1]**	**HKA·3-HBA**	**HKA (ZZZFMU01)**
Crystal system	monoclinic	orthorhombic	monoclinic	monoclinic	monoclinic
Space group	$P2_1/b$	$Pna2_1$	$P2_1/c$	$P2_1/c$	$P2_1/n$
a (Å)	5.493(2)	20.085(2)	9.6308(3)	20.2173(9)	3.8323(8)
b (Å)	23.893(9)	3.7591(4)	8.2989(2)	3.8191(3)	18.409(6)
c (Å)	4.943(2)	8.2934(10)	7.7396(2)	16.3559(7)	8.505(4)
α (°)	90	90	90	90	90
β (°)	90	90	98.716(3)	101.105(4)	96.56
γ (°)	105.70(3)	90	90	90	90
V (Å^3)	624.54	624.32	611.44	1239.22(12)	596
Z	4	4	4	4	4

Powder X-ray Diffraction, DSC and TGA

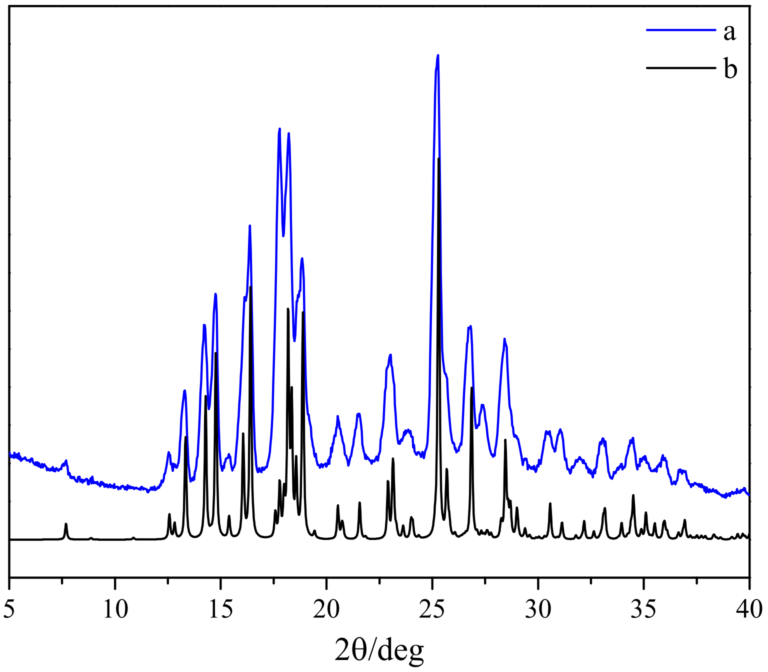

Figure S1. Comparison between the experimental XRD pattern for the slurry product (line a) of HKA with urotropine and the one calculated from single crystal data of HKA·urotropine (line b).

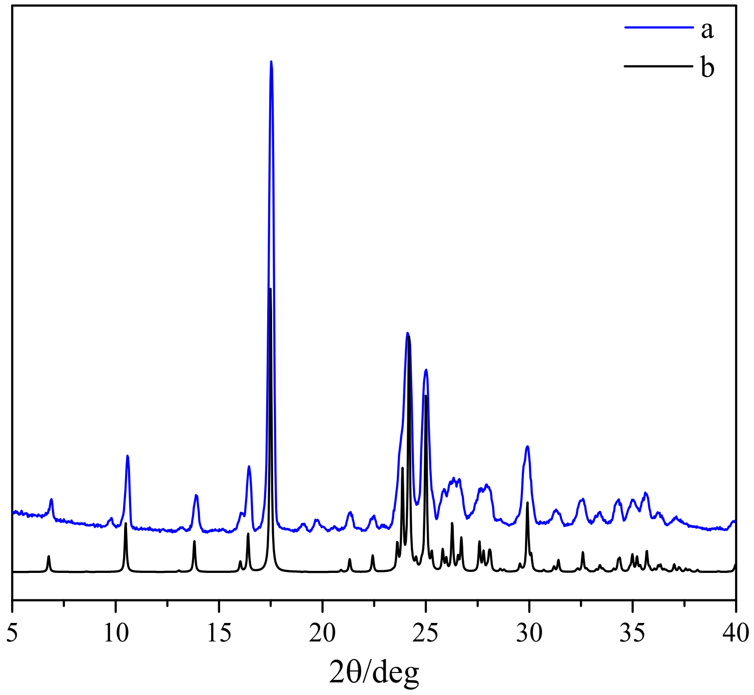

Figure S2. Comparison between the experimental XRD pattern for the ball milling product (line a) of HKA with 4-pyridone and the one calculated from single crystal data of HKA·4-pyridone (line b).

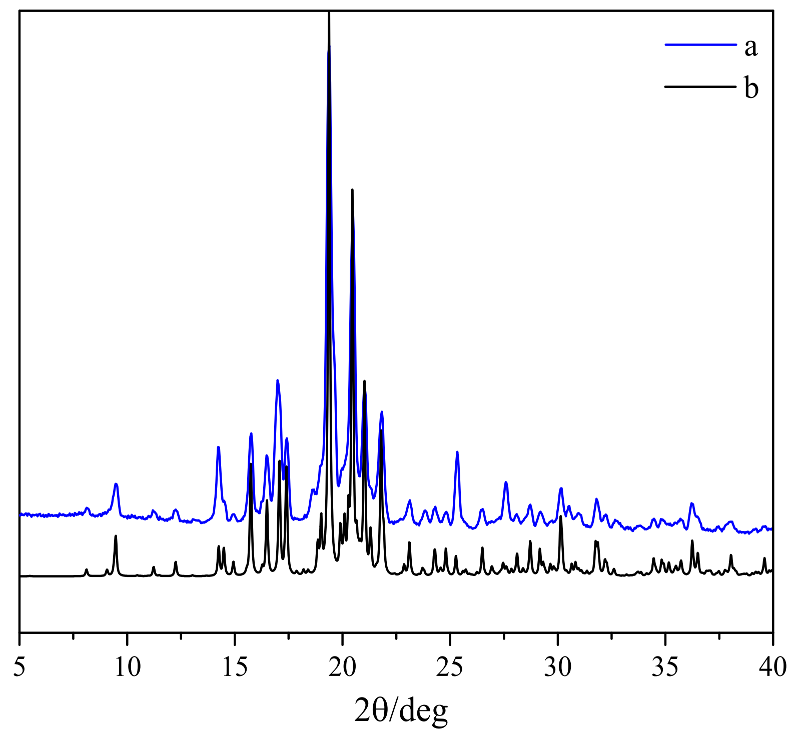

Figure S3. Comparison between the experimental XRD pattern for the manual grinding product (line a) of HKA with DABCO and the one calculated from single crystal data of HKA·DABCO (line b).

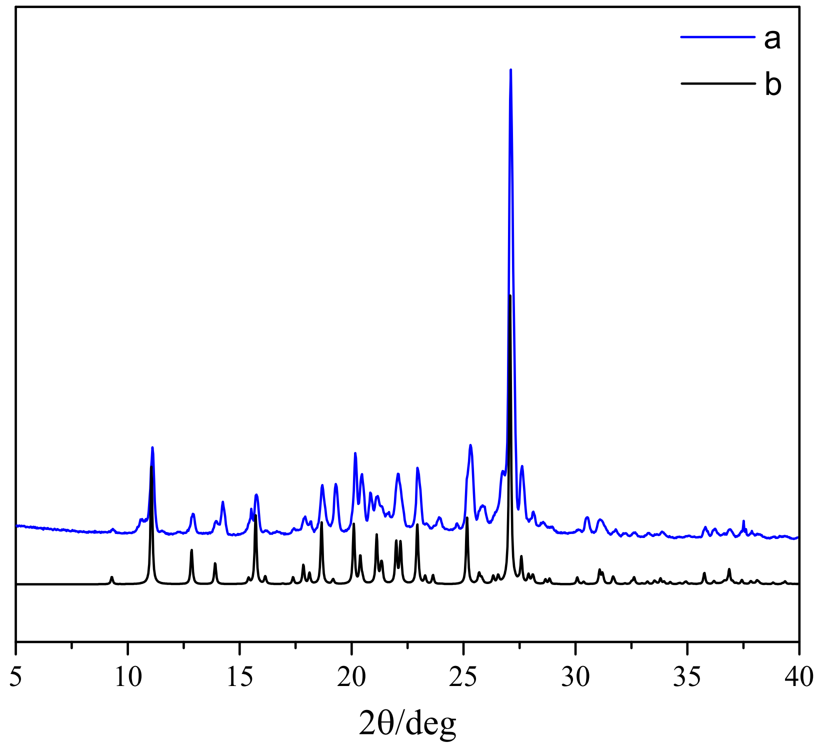

Figure S4. Comparison between the experimental XRD pattern for the ball milling product (line a) of HKA with Imidazole and the one calculated from single crystal data of HKA·imidazole (line b).

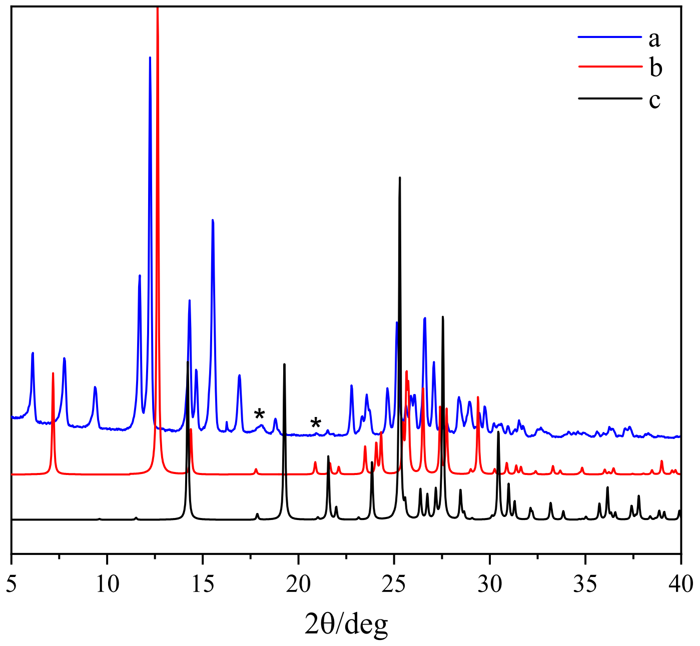

Figure S5. Comparison between the experimental XRD pattern for the product of the slurry in ethyl acetate (line a) of a 1:1 HKA:theophylline stoichiometric mixture and the patterns of the reagents theophylline (line b) and HKA (line c). Asterisks indicate traces of the reagents.

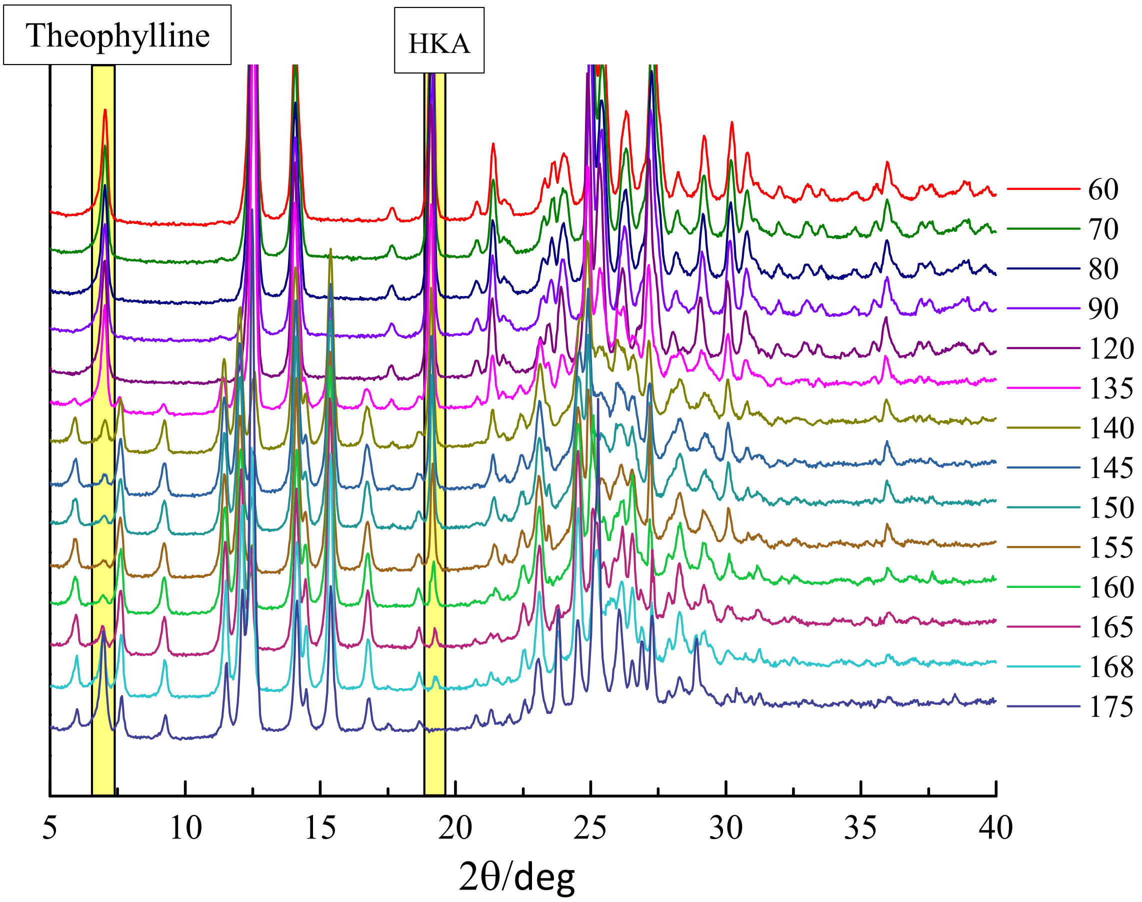

Figure S6. Variable temperature XRD patterns for the HKA:Theophylline 4:3 physical mixture. The

complex formation starts at ca. 135 °C, though with a slow kinetics: the unreacted kojic acid still present around 155 °C decomposes and theophylline becomes more evident. The complex is still present at 175 °C.

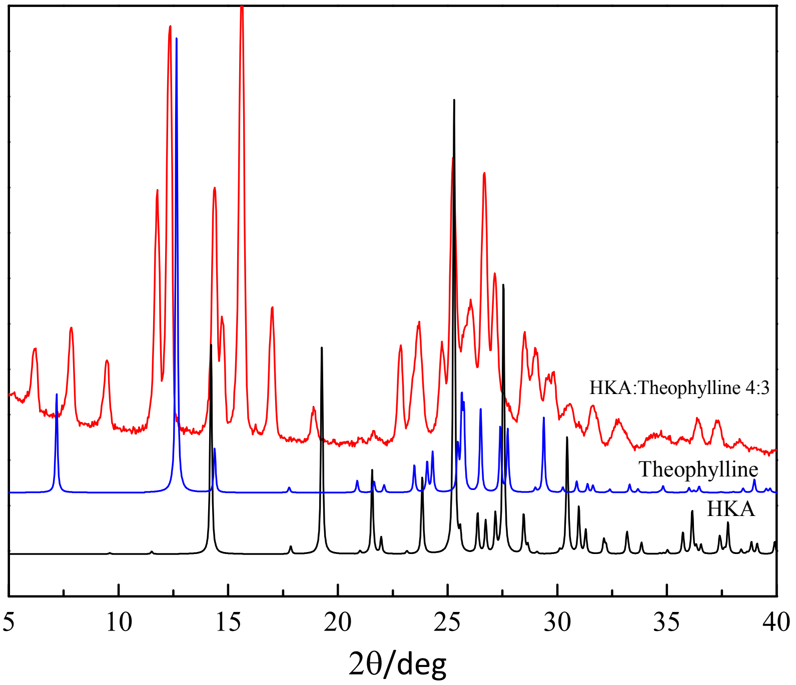

Figure S7. Comparison between the experimental XRD patterns of the HKA:theophylline 4:3 mixture, kept for one night at 140 °C, and the two reagents theophylline and HKA. Complex formation is complete, and no peaks of the reagents can be detected. The 4:3 stoichiometric ratio is thus confirmed.

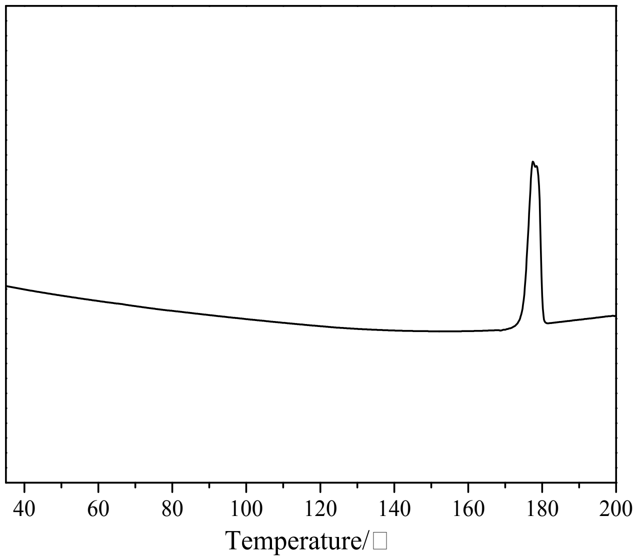

Figure S8. DSC trace of the crystalline complex $(HKA)_4{\cdot}(Theophylline)_3$ *after annealing the physical mixture for 16 h at 140 °C.*

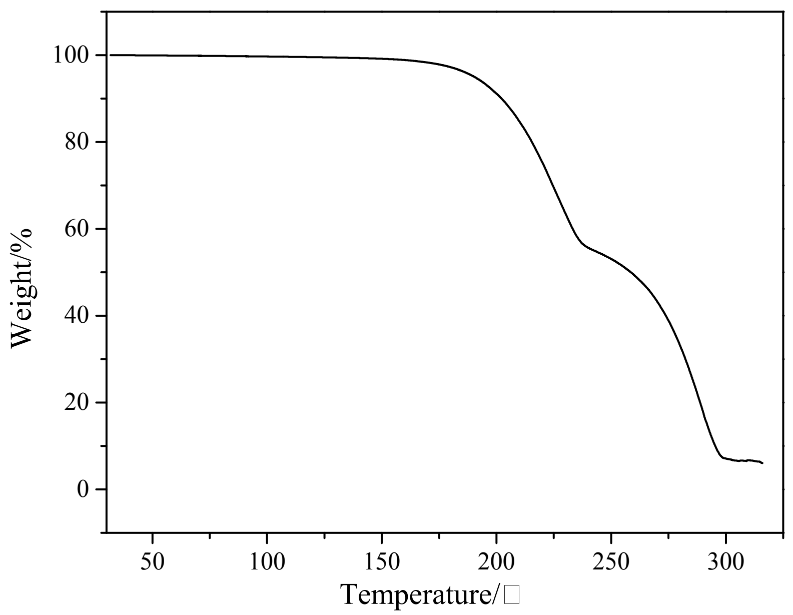

Figure S9. TGA trace of the crystalline complex $(HKA)_4{\cdot}(Theophylline)_3$

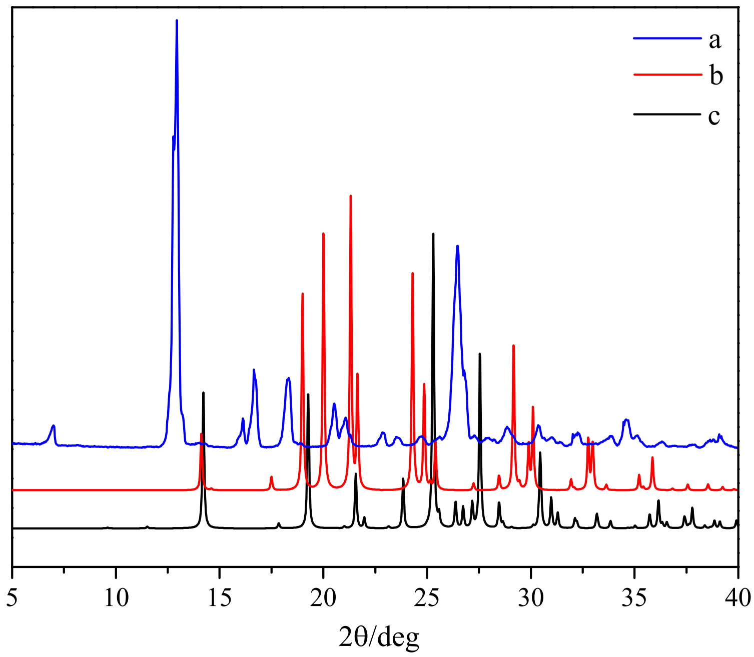

Figure S10. Comparison between the experimental XRD pattern for the ball milling product (line a) of HKA with 4-aminopyridine and the patterns of the reagents 4-aminopyridine (line b) and HKA (line c).

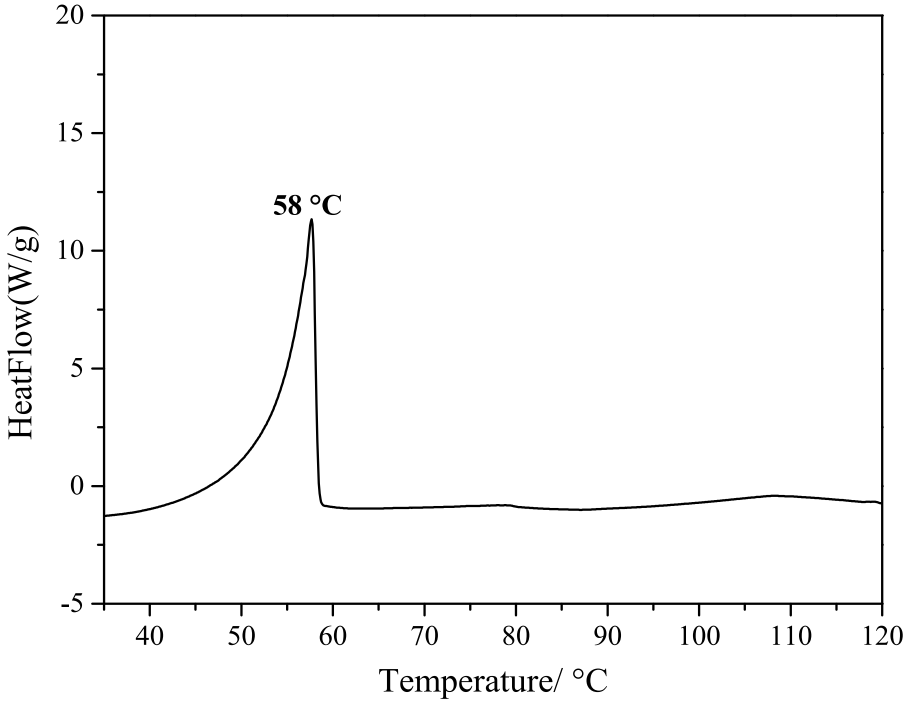

Figure S11. DSC trace of the crystalline complex HKA·4-aminopyridine.

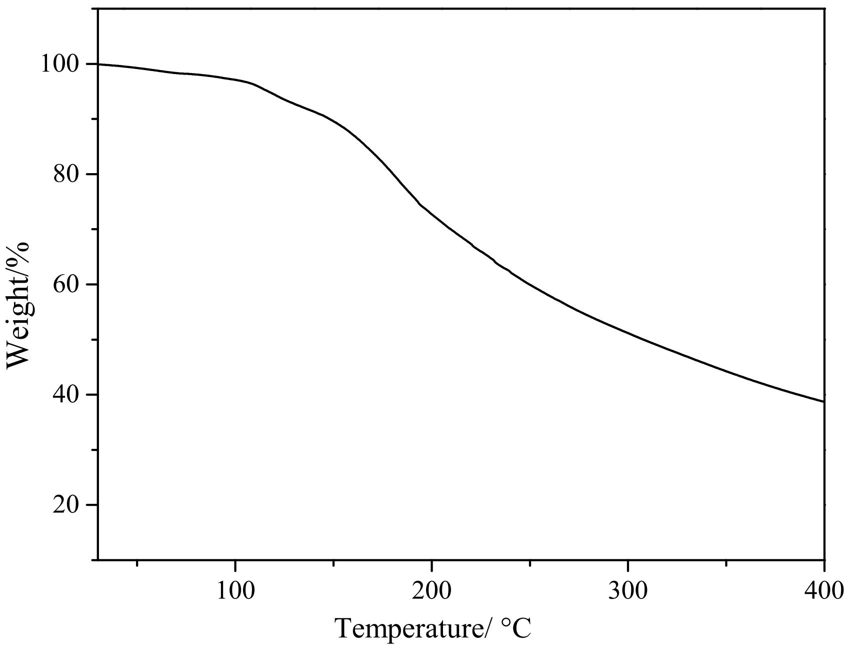

Figure S12. TGA trace of the crystalline complex HKA·4-aminopyridine.

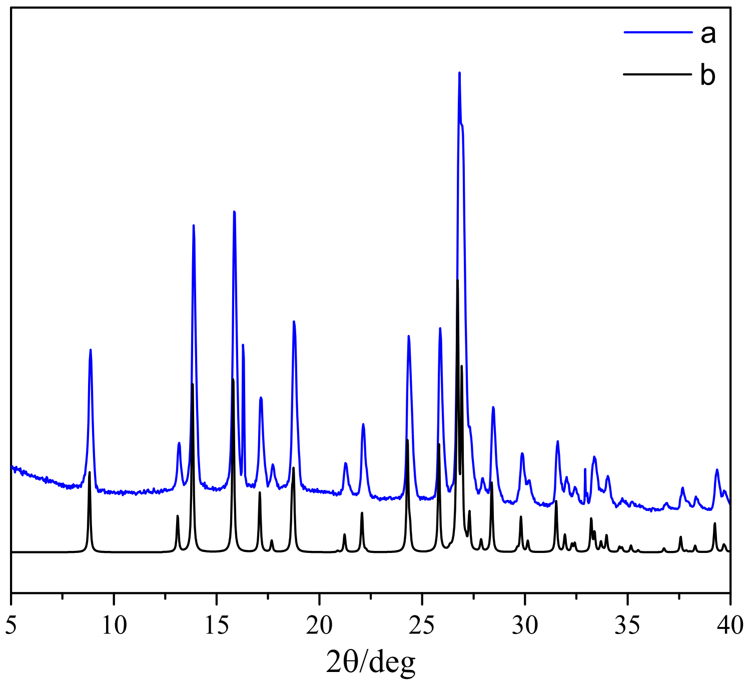

Figure S13. Comparison between the experimental XRD pattern for the ball milling product (line a) of HKA with piperazine and the one calculated from single crystal data of HKA·piperazine (line b).

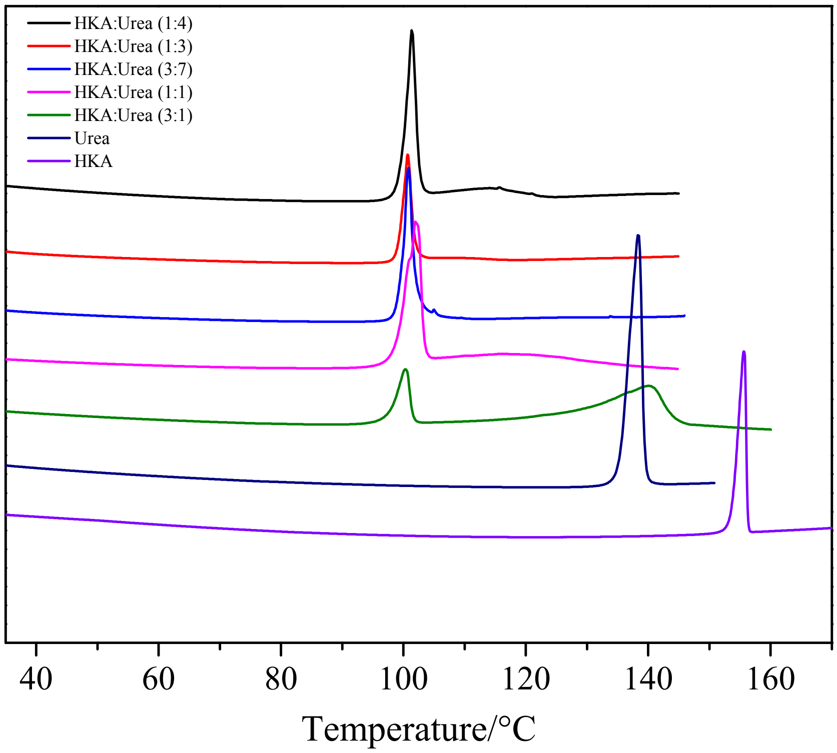

Figure S14. DSC trace of the eutectic mixtures of HKA:Urea system.

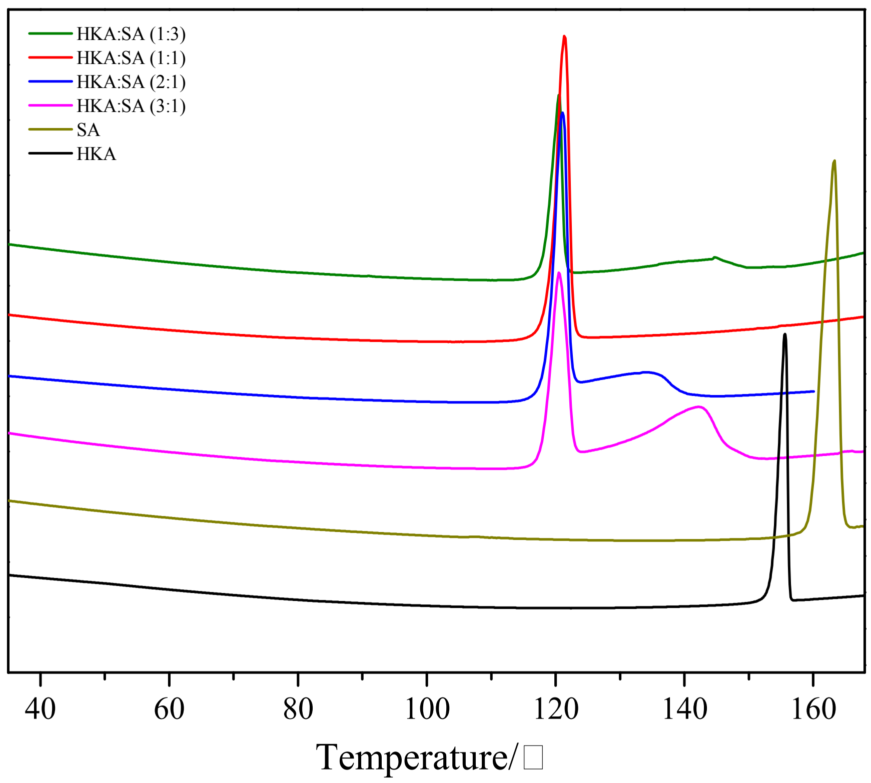

Figure S15. DSC traces of the eutectic mixtures of HKA:salicylic acid system.

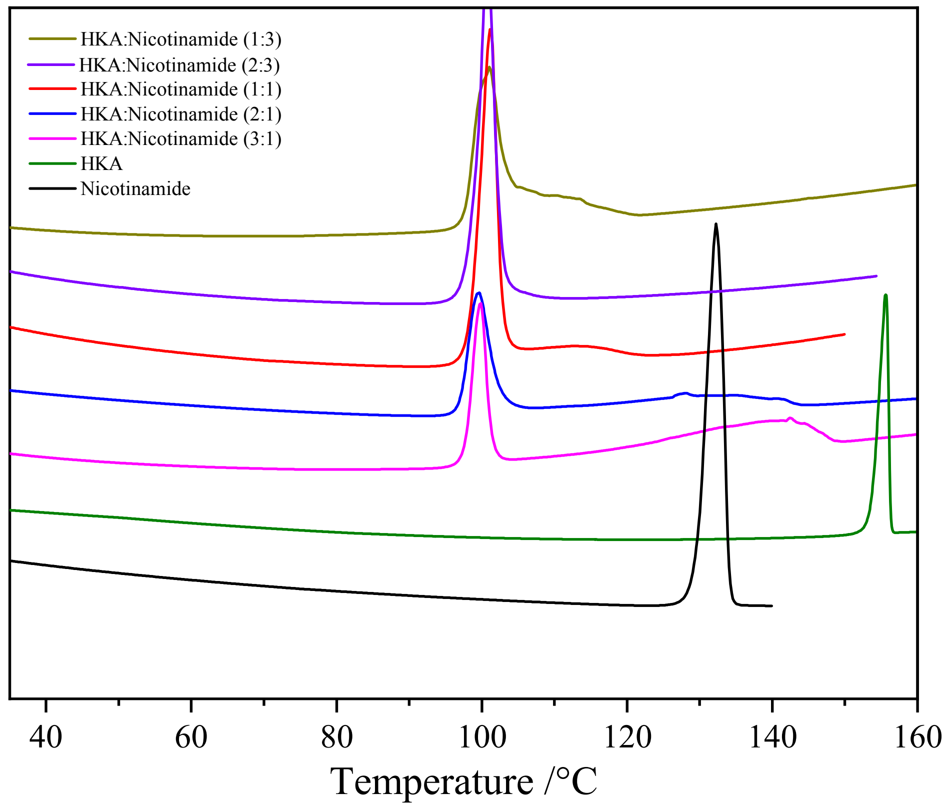

Figure S16. DSC traces of the eutectic mixtures of HKA:Nicotinamide system.

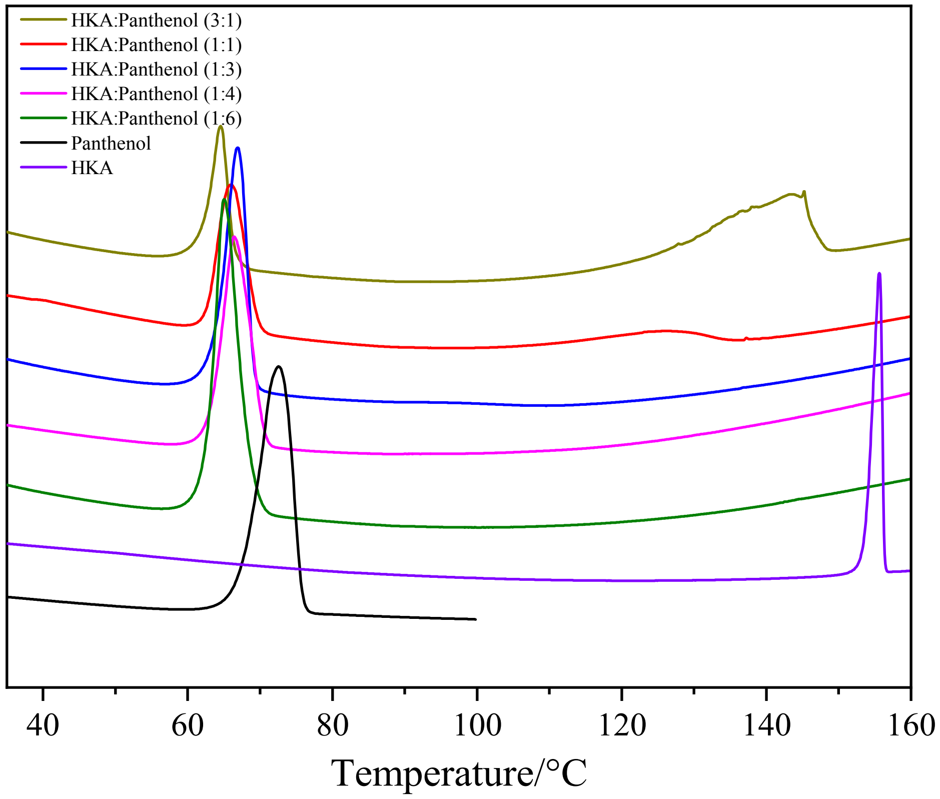

Figure S17. DSC traces of the eutectic mixtures of HKA:Panthenol system.

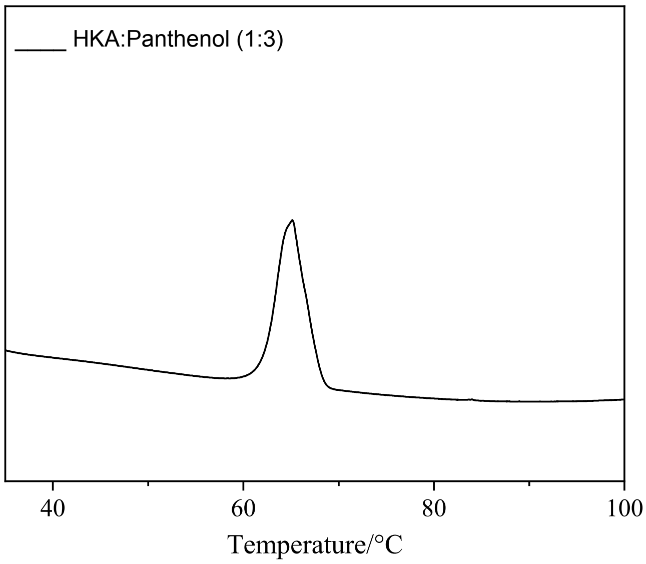

Figure S18. DSC trace of the 1:3 eutectic mixtures for the HKA:Panthenol system, repeated at a slower heating rate (2 °C/min). (Onset temperature 62 °C).

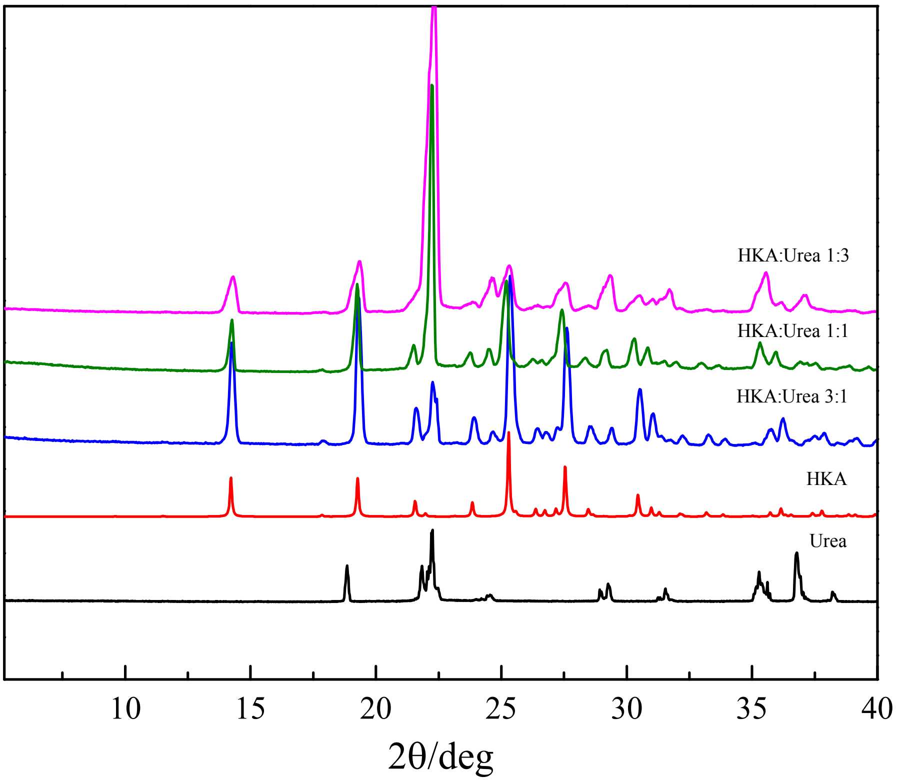

Figure S19. XRD patterns of the HKA-Urea system.

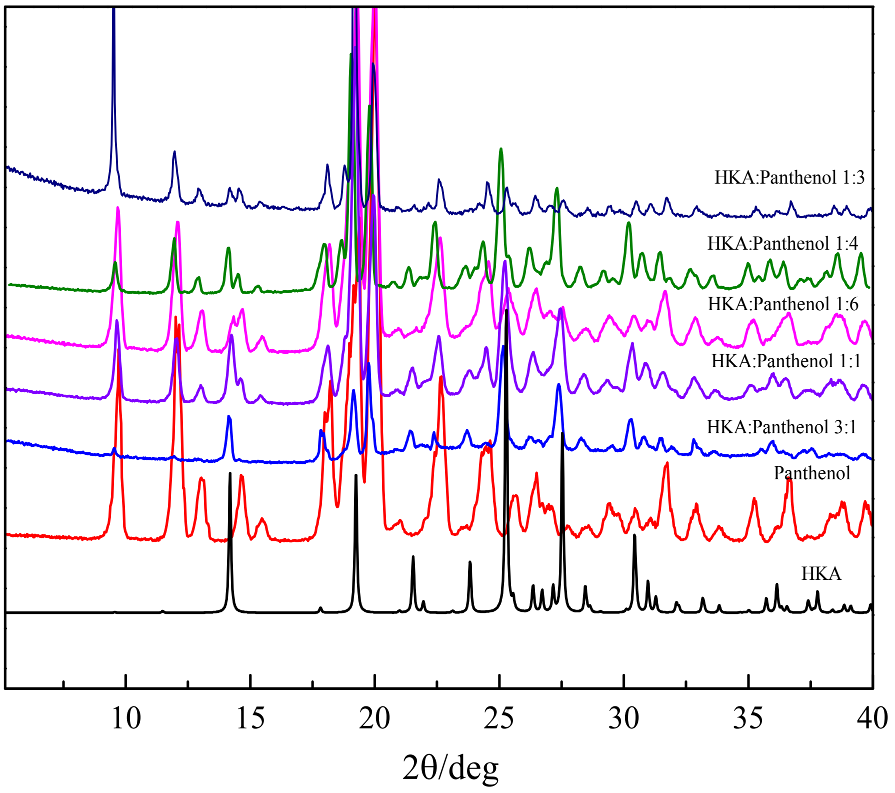

Figure S20. XRD patterns of the HKA-Panthenol system.

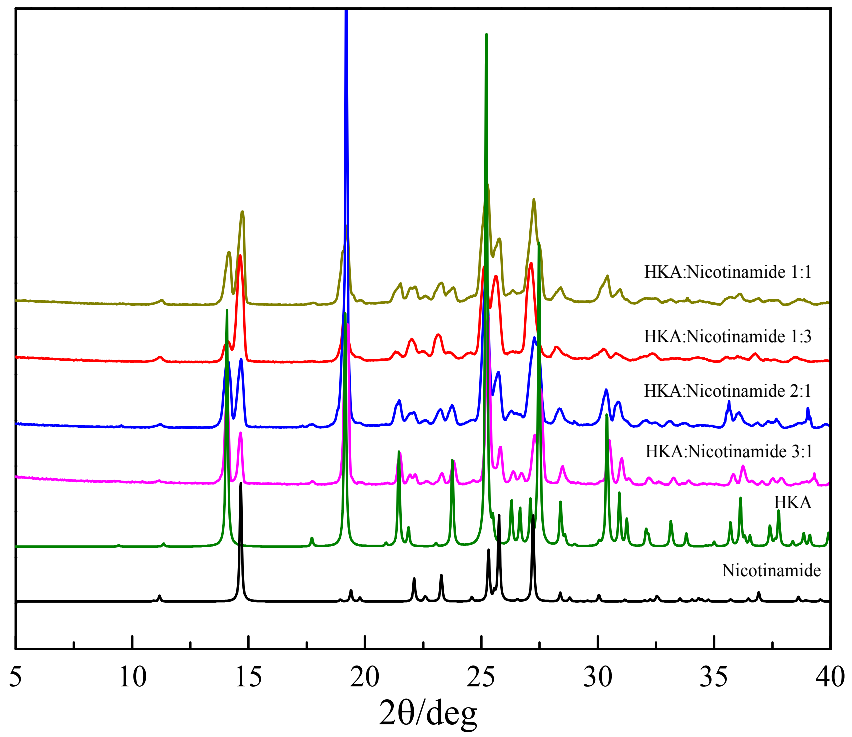

Figure S21. XRD patterns of the HKA-Nicotinamide system.

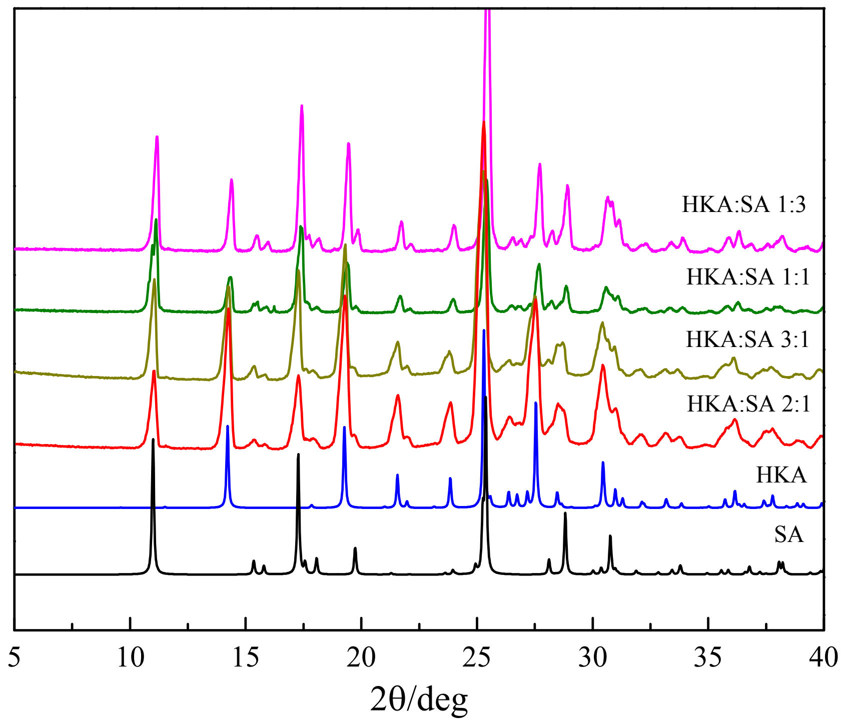

Figure S22. XRD patterns of the HKA-Salicylic acid system.

Co-former search (CSD)

Table S3. CSD Search for suitable co-formers of kojic acid (HKA) via Molecular Complementarity. The results are ordered according to decreasing *hit rate* %.

Hit Rate %	Co-former	Conformer* ZZZFMU01_00001	Conformer* ZZZFMU01_00002	Conformer* ZZZFMU01_00003
100	(+)-camphoric_acid	PASS	PASS	PASS
100	1-hydroxyethylidene-1,1-diphosphonic_acid	PASS	PASS	PASS
100	2-amino-5-methylbenzoic_acid	PASS	PASS	PASS
100	3-methylpyridine	PASS	PASS	PASS
100	4-acetamidobenzoic_acid	PASS	PASS	PASS
100	4-aminobenzoic_acid	PASS	PASS	PASS
100	4-hydroxybenzoic_acid	PASS	PASS	PASS
100	D-glucuronic_acid	PASS	PASS	PASS
100	D-pantothenol	PASS	PASS	PASS
100	EDTA	PASS	PASS	PASS
100	L-arginine	PASS	PASS	PASS
100	L-aspartic_acid	PASS	PASS	PASS
100	L-glutamic_acid	PASS	PASS	PASS
100	L-glutamine	PASS	PASS	PASS
100	L-glutathione	PASS	PASS	PASS
100	L-leucine	PASS	PASS	PASS
100	L-mandelic_acid	PASS	PASS	PASS
100	L-methionine	PASS	PASS	PASS
100	L-phenylalanine	PASS	PASS	PASS
100	L-proline	PASS	PASS	PASS
100	L-serine	PASS	PASS	PASS
100	L-tartaric_acid	PASS	PASS	PASS
100	L-tryptophan	PASS	PASS	PASS
100	L-tyrosine	PASS	PASS	PASS
100	N-ethylacetamide	PASS	PASS	PASS
100	acesulfame	PASS	PASS	PASS
100	acetic_acid	PASS	PASS	PASS
100	acetophenone_oxime	PASS	PASS	PASS
100	acetylenedicarboxylic_acid	PASS	PASS	PASS
100	adipic_acid	PASS	PASS	PASS
100	benzoic_acid	PASS	PASS	PASS
100	biotin	PASS	PASS	PASS
100	caprolactam	PASS	PASS	PASS
100	citric_acid	PASS	PASS	PASS
100	ethylparaben	PASS	PASS	PASS

100	fumaric_acid	PASS	PASS	PASS
100	gentisic_acid	PASS	PASS	PASS
100	glutaric_acid	PASS	PASS	PASS
100	glycine	PASS	PASS	PASS
100	glycine (zwitterionic)	PASS	PASS	PASS
100	glycolic_acid	PASS	PASS	PASS
100	hippuric_acid	PASS	PASS	PASS
100	hydrocinnamic_acid	PASS	PASS	PASS
100	imidazole	PASS	PASS	PASS
100	isonicotinamide	PASS	PASS	PASS
100	ketoglutaric_acid	PASS	PASS	PASS
100	lactose	PASS	PASS	PASS
100	maleic_acid	PASS	PASS	PASS
100	malic_acid	PASS	PASS	PASS
100	malonic_acid	PASS	PASS	PASS
100	methylparaben	PASS	PASS	PASS
100	monobutyrin	PASS	PASS	PASS
100	nicotinamide	PASS	PASS	PASS
100	oxalic_acid	PASS	PASS	PASS
100	phthalamide	PASS	PASS	PASS
100	piperazine	PASS	PASS	PASS
100	propylparaben	PASS	PASS	PASS
100	pyrazine	PASS	PASS	PASS
100	riboflavin	PASS	PASS	PASS
100	saccharin	PASS	PASS	PASS
100	sorbic_acid	PASS	PASS	PASS
100	succinic_acid	PASS	PASS	PASS
100	t-butylhydroxyanisole	PASS	PASS	PASS
100	theophylline	PASS	PASS	PASS
100	valerolactam	PASS	PASS	PASS
100	xanthine	PASS	PASS	PASS
67	(-)-camphorsulfonic_acid	FAIL	PASS	PASS
67	apigenin	PASS	FAIL	PASS
67	cholic_acid	PASS	PASS	FAIL
67	hesperetin	FAIL	PASS	PASS
67	thymidine	FAIL	PASS	PASS
33	D-alanine	FAIL	PASS	FAIL
33	L-lactic_acid	FAIL	PASS	FAIL
33	alitame	FAIL	PASS	FAIL
33	capsaicin	FAIL	PASS	FAIL
33	lactobionic_acid	FAIL	PASS	FAIL
33	maltitol	FAIL	PASS	FAIL
33	mannitol	FAIL	PASS	FAIL
33	pamoic_acid	FAIL	PASS	FAIL
0	D-alanine (zwitterionic)	FAIL	FAIL	FAIL

0	L-arginine (zwitterionic)	FAIL	FAIL	FAIL
0	L-aspartic_acid (zwitterionic)	FAIL	FAIL	FAIL
0	L-aspartic_acid	FAIL	FAIL	FAIL
0	L-glutamic_acid (zwitterionic)	FAIL	FAIL	FAIL
0	L-glutamic_acid	FAIL	FAIL	FAIL
0	L-glutamine (zwitterionic)	FAIL	FAIL	FAIL
0	L-glutathione (zwitterionic)	FAIL	FAIL	FAIL
0	L-leucine (zwitterionic)	FAIL	FAIL	FAIL
0	L-methionine (zwitterionic)	FAIL	FAIL	FAIL
0	L-phenylalanine (zwitterionic)	FAIL	FAIL	FAIL
0	L-proline (zwitterionic)	FAIL	FAIL	FAIL
0	L-serine (zwitterionic)	FAIL	FAIL	FAIL
0	L-tryptophan (zwitterionic)	FAIL	FAIL	FAIL
0	L-tyrosine (zwitterionic)	FAIL	FAIL	FAIL
0	azelaic_acid	FAIL	FAIL	FAIL
0	folic_acid	FAIL	FAIL	FAIL
0	methanesulfonic_acid	FAIL	FAIL	FAIL
0	pimelic_acid	FAIL	FAIL	FAIL
0	suberic_acid	FAIL	FAIL	FAIL
0	t-butylamine	FAIL	FAIL	FAIL
0	triphenylacetic_acid	FAIL	FAIL	FAIL
0	urea	FAIL	FAIL	FAIL

* See Figure S23

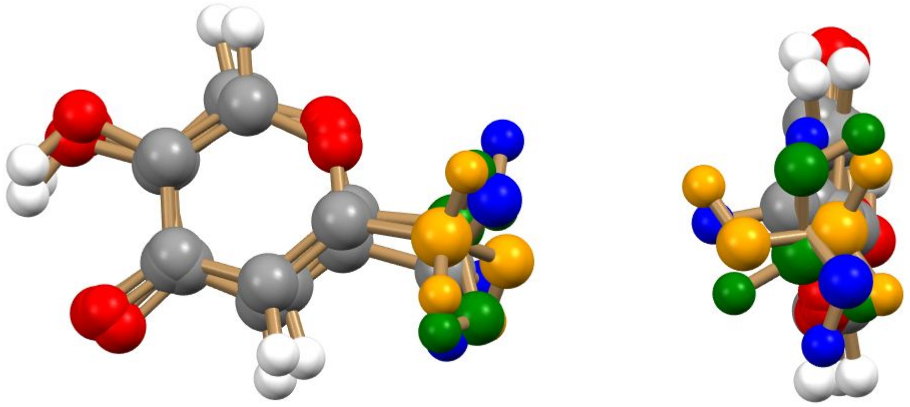

Figure S23. Superimposition of the three conformations generated via the "Conformer Generator" utility (CSD-Materials module in the program Mercury), starting from the coordinated deposited in the Cambridge Structural Database for kojic acid (refcode (ZZZFMU01). It can be seen that the only difference is in the orientation of the -CH_2OH groups (here shown in yellow, green and blue).

Table S4. Multi-component hydrogen-bond propensity screen results. All coformers in the list were used for co-crystallization attempts with HKA. In green the co-formers that yielded co-crystals.

Rank	Component B	Multi-component score	Max interaction	Max A:B or B:A propensity	Max A:A propensity	Max B:B propensity
1	pyrazine	0.24	A:B	0.79	0.55	0.0
2	4-aminobenzoic_acid	0.14	B:A	0.77	0.63	0.61
3	L-aspartic_acid	0.05	B:A	0.63	0.57	0.58
4	L-glutamic_acid	0.05	B:A	0.62	0.57	0.56
5	riboflavin	0.05	A:B	0.78	0.57	0.73
6	L-arginine	0.04	B:A	0.74	0.55	0.7
7	L-methionine	0.04	B:A	0.62	0.55	0.58
8	L-serine	0.02	B:A	0.65	0.56	0.63
9	L-tartaric_acid	0.02	B:A	0.61	0.59	0.57
10	D-alanine	0.01	B:A	0.64	0.55	0.62
11	xanthine	0.01	A:B	0.69	0.6	0.68
12	imidazole	0.0	B:A	0.62	0.62	0.61
13	piperazine	0.0	B:A	0.61	0.6	0.53
14	saccharin	-0.0	A:A	0.61	0.62	0.43
15	glycolic_acid	-0.01	A:A	0.65	0.66	0.63
16	oxalic_acid	-0.01	A:A	0.61	0.61	0.46
17	glycine	-0.02	B:B	0.64	0.55	0.66
18	acetic_acid	-0.03	A:A	0.54	0.57	0.45
19	adipic_acid	-0.03	A:A	0.62	0.66	0.56
20	EDTA	-0.03	A:A	0.58	0.61	0.53
21	theophylline	-0.03	A:A	0.54	0.57	0.46
22	glutaric_acid	-0.04	A:A	0.62	0.66	0.55
23	L-tyrosine	-0.04	A:A	0.64	0.68	0.48
24	malonic_acid	-0.04	A:A	0.62	0.66	0.56
25	nicotinamide	-0.04	B:B	0.77	0.54	0.81
26	succinic_acid	-0.04	A:A	0.62	0.66	0.55
27	citric_acid	-0.06	A:A	0.62	0.68	0.52
28	fumaric_acid	-0.06	A:A	0.6	0.66	0.51
29	L-glutamine	-0.06	B:B	0.68	0.55	0.73
30	maleic_acid	-0.06	A:A	0.59	0.65	0.51
31	L-proline	-0.07	A:A	0.5	0.57	0.43
32	sorbic_acid	-0.07	A:A	0.59	0.65	0.5
33	benzoic_acid	-0.09	A:A	0.52	0.61	0.38
34	3-hydroxybenzoic_acid Form I	-0.11	A:A	0.62	0.73	0.44

35	L-tyrosine- zwitterion	-0.07	B:B	0.91	0.57	0.98
36	Alanine-zwitterion	-0.08	B:B	0.9	0.57	0.98
37	glycine-zwitterion	-0.08	B:B	0.9	0.57	0.98
38	L-proline-zwitterion	-0.08	B:B	0.87	0.52	0.95
39	Serine-zwitterion	-0.08	B:B	0.91	0.57	0.98
40	L-arginine- zwitterion	-0.09	B:B	0.88	0.59	0.96
41	L-glutamine- zwitterion	-0.09	B:B	0.88	0.56	0.97
42	L-methionine-zwitterion	-0.09	B:B	0.89	0.52	0.98
43	L-aspartic_acid-zwitterion	-0.1	B:B	0.87	0.55	0.97
44	L-glutamic_acid-zwitterion	-0.1	B:B	0.87	0.55	0.97

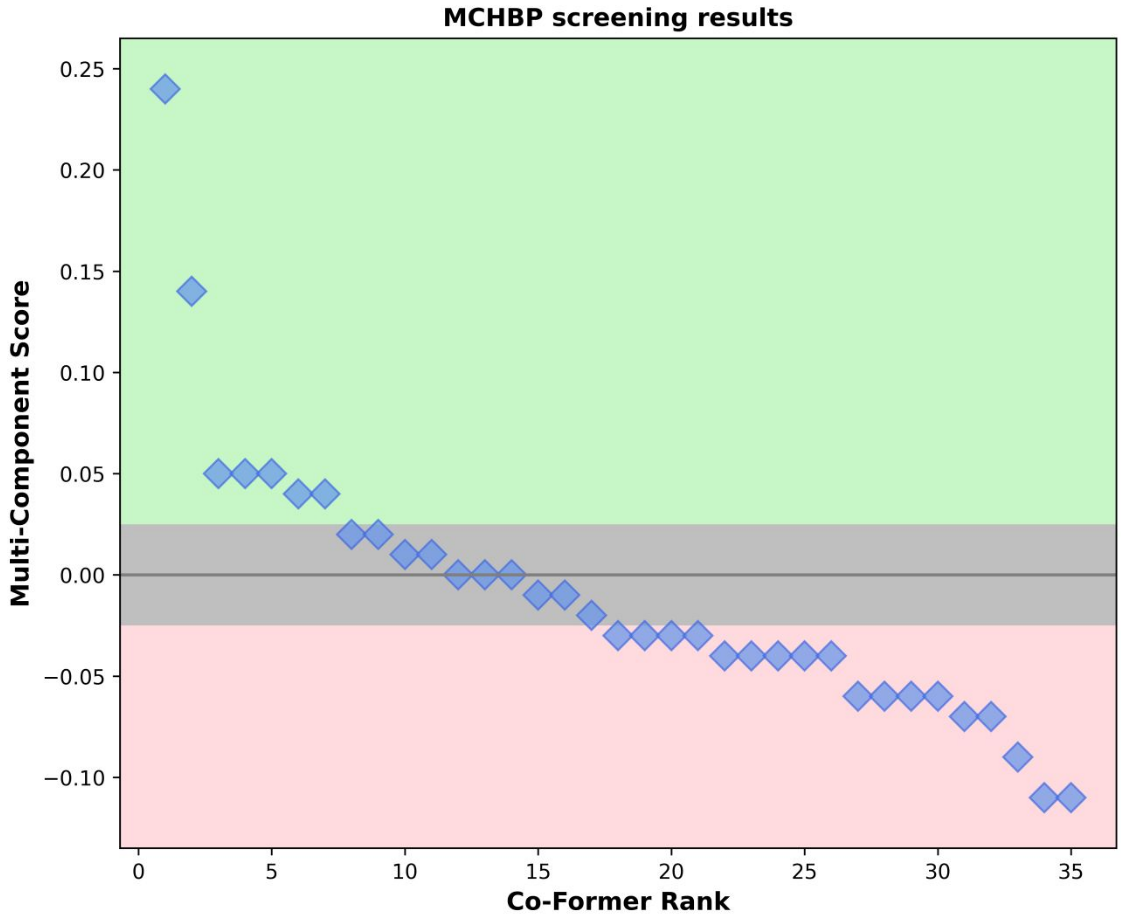

Figure S24. Multi-component hydrogen-bond propensity screen results

Table S5. List of all co-formers and synthetic methods used for co-crystallization attempts with HKA*.

Kojic acid	**Solution**	**Result**	**Slurry**	**Result**	**Ball milling**	**Result**
Urea	Water/EtOH/MeOH(1:1)(1:2)	×	EAC(1:1)	×	Water/EtOH(1:1)(1:2)	×
Salicylic acid	Water/acetic acid/water+EtOH(1:1)	×	EAC(1:1)(1:2)	×	Water(1:1)	×
Xanthine			EAC(1:1)	×	Water+EtOH(1:1)	×
Riboflavin	DMSO+Water(1:1)	×			Water(1:1)	×
L-tartaric_acid	Water+EtOH/EtOH(1:1)	×			Water+ EtOH(1:1)	×
Acetic acid	solution	×			LAG	
Panthenol	MeOH(1:1)(1:2)(2:1)	×	NPA(1:1)(1:2)(1:3)	×	Water(1:1)	×
Caffeine	Water/ACN+MeOH(1:1)	×	Acetone(1:1)	×	Water/ACN/ACN+MeOH(1:1)(1:2)(2:1)	×
Theophylline	Water/ACN+MeOH/EtOH/MeOH(1:1) (1:2)(2:1)(4:3)	Water- ×	EAC/EtOH/MeOH/Acetone(1:1)(4:3)	√	Water/ACN/MeOH/ACN+MeOH (1:1)(1:2)(1:3)(2:3)(3:1)(3:2)(4:3)(8:1)	Water-×
Theobromine	DMSO+Water(1:1)	NOT yet	EAC(1:1)	×	Water(1:1)	×
Quinoxaline	Water/MeOH(1:1)	×	Melt quinoxaline+HKA(3:1)(1:1)	×	Water/water+ACN(1:1)(2:1)(1:2)	×
Nicotinamide	Water+EtOH/ACN(1:1)	×	Acetone+EG(1:1)	×	Water/ACN-/EG(1:1)	×
Piperazine	ACN+MeOH/Choloroform(1:1)	Choloroform-×			NG/MeOH(1:1)	√
Imidazole	MeOH/EtOH(1:1)	√			Water/MeOH(1:1)	×
4-aminopyridine	Water/MeOH(1:1)(1:2)	×			Water(1:1)	×
Urotropine	Water(1:1)	√	EAC/Water+EAC/Water+EtOH(1:1)	EAC/Water+EAC-×	Water/MeOH/NG(1:1)	×
4-Pyridone	Water(1:1)	√			Water(1:1)	√
DABCO	EtOH(1:1)	√			Water/MeOH/NG/EtOH(1:1)	√
3-HBA	Water+EtOH/Water(1:1)(3:7)(2:3)(3:2)(7:3)(1:4)(4:1)	Water ×			Water/EtOH/Water+EtOH(1:1)	×
L-arginine	Water/EtOH(1:1)(1:2)	×	EtOH(1:1)(1:2)	×	EtOH/water+EtOH(1:1)(2:1)	×

Pyrazine	ACN+MeOH(1:1)	×	NPA(1:1)	×	ACN(1:1)	×
Adipic acid					Water(1:1)	×
EDTA					Water(1:1)	×
Glutaric acid					Water/EtOH(1:1)	×
Malonic acid					Water/EtOH/MeOH(1:1)	×
Succinic acid					Water+EtOH(1:1)	×
Citric acid					Water(1:1)	×
Fumaric acid					Water(1:1)	×
Maleic acid					Water(1:1)	×
Sorbic acid					Water(1:1)	×
Benzoic acid					Water(1:1)	×
D-alanine					Water(1:1)	×
L-glutamine					Water/EtOH(1:1)	×
L-serine					Water(1:1)	×
L-glutamic acid					Water(1:1)	×
L-glycine					Water(1:1)	×
L-methionine					Water(1:1)	×
L-proline					Water(1:1)	×
L-tyrosine					Water(1:1)	×
4-aminobenzoic acid					Water(1:1)	×
L-aspartic_acid					Water(1:1)	×
Saccharin					Water(1:1)	×
Glycolic acid					Water(1:1)	×
Oxalic acid					Water(1:1)	×

*MeOH-methanol; EtOH-ethanol; NPA-1-propanol; ACN-acetonitrile; EAC-ethyl acetate; NG- Neat grinding; LAG- Liquid assisted grinding; EG- ethylene glycol; the ratio is(HKA:CO-former)

Pairwise intermolecular interactions

Table S6. Pairwise intermolecular interactions[a] seen in HKA, coformers and cocrystals.

Compound	Interaction	n	E_E	E_P	E_D	E_R	E_{tot}^b
	Notes		kJ mol^{-1}				
**HKA**	O-H···O	2	-73.8	-18.8	-12.5	94	-44.8
	O-H···O	2	-43.2	-7.5	-10.8	57	-25.4
		2	-7.6	-2.9	-36.5	32.7	-21.8
	2x C-H···O	1	-21.2	-3.2	-11.2	28.1	-17.2
		1	-4.7	-0.8	-15.1	9.4	-12.9
		1	0.3	-0.7	-14	7.3	-7.8
		2	-0.4	-2	-9.6	4.2	-7.7
		1	-2.1	-0.7	-8.8	5.3	-7.2
		2	0	-0.2	-1.3	0	-1.2
**3-HBA**	O-H···O	2	-70	-16.1	-12	90.1	-40.7
	O-H···O	2	-57.5	-11.7	-10.6	76.9	-31.1
	π···π	2	-2.6	-1.4	-36	24.4	-20
		2	-6.4	-0.7	-12.5	14.9	-8.9
		2	-0.7	-1.1	-9.2	2.6	-7.9
		2	-2.2	-0.3	-10.3	7.1	-7.1
		2	-0.1	-0.1	-1.6	0	-1.5
		2	0.5	-0.1	-1.6	0	-0.9
**Cocrystal**	O-H···O	2	-78.3	-18.9	-10.8	97.9	-45.8
**HKA 3-HBA**	O-H···O	2	-61.4	-15.2	-13.9	76	-41.4
	O-H···O	2	-47.1	-7.8	-9.6	57.8	-28.2
	O-H···O	2	-56.8	-12.1	-10.3	81.3	-27.8
	π···π	2	-7.1	-2.9	-36.2	31.1	-21.9
	π···π	2	-0.1	-1	-31.7	17.8	-17.4
	2x C-H···O	1	-29.2	-4.2	-10.8	42.2	-17.2
		2	-9.1	-0.8	-18	24.2	-11
		2	-5.1	-1.6	-7.8	4.5	-10.6
		2	0.9	-1.7	-9.8	3	-7
		2	1.1	-0.7	-12.7	6.2	-6.6
		1	-1.9	-0.4	-9.8	7.7	-6.1
		2	-1.3	-0.5	-6.1	3.3	-5
		2	-1.5	-0.3	-3.6	0.6	-4.5
		1	-1.4	-0.1	-1.4	0	-2.8
		1	-0.1	-0.1	-1.5	0	-1.5
**Imidazole**	N-H···O	2	-69.3	-17.2	-8.6	79.2	-44.6
	π···π	1	-10.9	-1.7	-12.4	5.5	-20.2
	C-H···π	2	-8.5	-1.3	-13.1	9.3	-15.7
		2	-7.3	-2.7	-7.7	8.2	-11.3
		1	-2.8	-1.2	-6.5	4.2	-6.9
		2	0.5	-0.8	-6.2	3.9	-2.9

		1	2.5	-1.8	-7	3.9	-2.4
		2	0.5	-0.6	-4.5	3.8	-1.4
		1	1.9	-0.4	-1.4	0	0.5
**Cocrystal (Z'=2)**	O-H···N	2	-101.2	-25.9	-12.1	135.1	-53.2
**HKA imizazole**	N-H···O	2	-63.6	-16.1	-12.1	63.5	-50.5
	N-H···O	2	-65.7	-16.4	-12.4	68.8	-49.9
	O-H···N	2	-105.6	-26.9	-12.7	150.7	-49.5
	O-H···O	2	-73.5	-18.3	-11.7	95.1	-42.7
	O-H···O	2	-68.8	-17	-12.1	87.5	-41.7
	π···π	1	-17.6	-4.7	-28.3	19.3	-34.8
	π···π	1	-13.4	-6	-22.5	12.8	-30.3
	C-H···π	2	-13.7	-2.6	-22.1	19.1	-23.9
	C-H···π	2	-9.9	-1.9	-17	14.9	-17.5
		2	-4.8	-1.5	-14.1	8.5	-13.1
		2	4.1	-4.5	-21	12.2	-9.7
		2	-3.5	-1.7	-8	4.9	-8.9
		2	-5.9	-0.7	-3.1	1.2	-8.6
		1	-9.7	-1	-13.4	23.5	-8.2
		2	-5.6	-0.5	-2.2	0.3	-8
		1	-8.8	-1	-13.7	22.9	-7.8
		2	-4.2	-1.3	-1.7	0	-6.8
		2	3.8	-3.5	-14.5	7.9	-6.3
		2	-1.6	-0.9	-3.1	0.3	-4.9
		2	-2.3	-1.9	-7.7	9.4	-4.7
		2	-0.7	-0.4	-7	4.6	-4.4
		2	-0.7	-0.9	-3.7	0.7	-4.2
		2	-0.3	-0.4	-6	3.1	-3.9
		2	5.1	-2.4	-14.8	9.4	-3.4
		2	2.4	-1.6	-6.7	3.2	-2.5
		2	3.1	-1.5	-5.2	2.9	-0.6
		2	7.6	-1.4	-4.2	1.5	4.2
		2	9	-0.8	-2.6	0.7	7.1
**Pyridone**	N-H···O	2	-71.6	-17.2	-6.5	67.6	-52.3
	π···π	1	-24.5	-3.5	-27.1	20.8	-39.2
	π···π	1	-23.5	-3.5	-23.4	14.9	-38.6
	C-H···O	2	-15.7	-4.7	-10.3	8.8	-23.6
	C-H···O/ C-H···π	2	-17.4	-5.3	-14.1	20.5	-22
		1	-3	-4.6	-9.9	8.5	-10
		1	0.4	-1.1	-8.4	5.1	-4.6
		2	0.6	-2.4	-10.2	14.3	-1.2
		1	1	-0.3	-0.7	0	0.3
		1	9.7	-1.9	-1.8	0.1	7.4
		1	12	-2.2	-2	0.2	9.4
		1	16.9	-3.1	-7.9	3	10.5
**Cocrystal**	O-H···O	2	-109.2	-28.8	-11.7	121.9	-71.6

**HKA Pyridone**	N-H···O	2	-82.5	-20.1	-13.4	92.6	-56.6
	O-H···O	2	-78.8	-21	-11.8	85.6	-56.3
	2x C-H···O	1	-33.4	-10.7	-14.6	40.3	-31.1
	HKA···HKA	1	-17	-3.9	-12.2	6	-27.8
	PYR···PYR	2	-15.5	-1.9	-10.2	7.9	-21.7
	PYR···PYR	2	-13.9	-1.6	-10.3	11.8	-17.6
		2	-4.2	-4.3	-29.8	32.9	-13.2
		1	-7.6	-1	-4.9	0.1	-12.9
		2	-4.1	-1.8	-7.6	3.7	-10
		2	-0.6	-3.4	-8.5	7.4	-6
		2	-1.8	-1.3	-1.7	0.1	-4.3
		2	3.7	-1.5	-3.6	0.6	0.1
		2	4	-1.2	-4.4	1.9	0.6
		1	7.5	-3.6	-17.1	22.1	4
		2	18.6	-3.6	-20.9	11.6	5.9
**DABCO**	2x C-H···N	6	-9.9	-2.4	-19.3	17.8	-18
		6	-1.6	-0.2	-16.2	11.1	-9.1
		6	-0.3	0	-1.3	0	-1.4
**Cocrystal (Z'=3)**	O-H···N	2	-77.1	-17.9	-19.5	101.4	-49.1
**HKA DABCO**	O-H···N	2	-69.5	-18.5	-17	85.7	-49
(orientation 1)	O-H···N	2	-54.8	-15.3	-17.2	57.5	-48.7
	O-H···N	2	-69.9	-16	-19.1	90.8	-46.3
	O-H···N	2	-69.7	-15.9	-19.3	92	-45.5
	O-H···N	2	-29.4	-10.5	-15.8	40.2	-27.8
	HKA···HKA	1	-13.4	-2.6	-11.7	9.6	-20.4
	C-H···π	2	-6	-0.6	-23.6	16.7	-17.1
	C-H···π	2	-4.2	-0.7	-21.2	11.7	-16.1
	C-H···π	2	-5.2	-0.8	-21	13.9	-15.8
	DABCO···DACBO	2	-7.2	-3.5	-13	9.8	-15.4
	C-H···π	2	-4	-1	-18	8.8	-15.1
	C-H···O	2	-16.3	-3.5	-8	19.1	-15
		2	-1.8	-0.3	-20.2	11.2	-12.9
		2	-1.6	-0.3	-17.1	7.6	-12.2
		2	-1.7	-0.3	-16.9	7.5	-12.2
		2	-2	-1.3	-15.2	8.8	-10.8
		2	-5.9	-2.6	-7.7	6.7	-10.7
		2	-4.2	-2.5	-9.1	6.7	-10.1
		2	-3.8	-0.7	-10.8	6.3	-10
		2	-3.3	-0.5	-9.5	3.8	-9.8
		2	-2.6	-0.6	-9.8	3.5	-9.7
		2	-2.4	-0.5	-9.7	3.2	-9.6
		2	-4	-0.5	-10.4	6.8	-9.4
		2	-2.1	-0.3	-11.1	5	-9.1
		2	-0.3	-1.6	-15.1	9.3	-8.9
		2	-2.1	-0.4	-9.4	3	-8.9

		2	-3.2	-0.4	-10	6.2	-8.5
		2	-0.8	-0.2	-12.5	6.4	-7.9
		1	-0.7	-0.2	-13.5	7.8	-7.9
		2	-1.8	-1.1	-12.3	9.3	-7.6
		2	-0.5	-0.7	-9.2	2.8	-7.4
		2	-0.8	-0.4	-11.1	5.8	-7.3
		2	-2.1	-0.3	-6.9	2	-7.2
		2	-0.4	-1	-8.3	2.2	-7.1
		2	-0.7	-0.6	-9.6	5.5	-6.1
		2	-3.4	-0.4	-1.9	0.1	-5.5
		2	0	-0.9	-6.9	2.1	-5.4
		2	-1.2	-0.2	-5.5	1.6	-5.2
		2	-1.1	-0.2	-6.3	2.9	-5
		1	-1.1	-0.2	-4.8	1	-4.9
		2	-0.8	-0.2	-3.4	0.2	-3.8
		2	-0.6	-0.1	-2	0.1	-2.4
**Cocrystal (Z'=3)**	O-H···N	2	-89.6	-21.8	-18.7	126.5	-48.9
**HKA DABCO**	O-H···N	2	-73.3	-17.9	-17.4	95.1	-47.2
(orientation 2)	O-H···N	2	-74.7	-18.1	-17.7	99.4	-46.5
	O-H···N	2	-57.3	-16.1	-16.0	65.8	-45.7
	O-H···N	2	-66.2	-17.6	-15.3	83.9	-44.6
	O-H···N	2	-71.0	-18.5	-15.1	95.4	-42.9
	HKA···HKA	1	-13.3	-3.5	-13.3	19.1	-16.4
	C-H···π	2	-6.5	-0.6	-22.7	18.7	-15.5
	C-H···π	2	-5.4	-0.8	-21.0	15.3	-15.1
	DABCO···DACBO	2	-8.2	-4.1	-13.3	13.4	-14.9
	C-H···O	1	-17.9	-3.7	-7.5	22.1	-14.6
	C-H···π	2	-6.5	-1.0	-21.2	19.1	-14.3
	C-H···π	2	-4.2	-0.5	-18.9	11.9	-13.9
	C-H···π	2	-3.9	-1.0	-18.0	11.2	-13.6
		2	-10.3	-2.1	-10.6	15.1	-12.3
		2	-2.4	-0.3	-17.0	9.7	-11.5
		2	-7.8	-3.2	-7.7	9.2	-11.5
		2	-2.4	-0.3	-17.0	9.7	-11.5
		2	-3.5	-1.0	-9.2	2.4	-11.0
		2	-5.1	-2.9	-9.2	8.6	-10.3
		2	-2.8	-1.5	-15.8	12.2	-10.2
		2	-3.5	-0.5	-9.9	5.3	-9.5
		2	-3.1	-0.6	-9.9	6.1	-8.5
		2	-4.4	-0.5	-10.4	9.2	-8.4
		2	-2.1	-0.4	-9.4	4.0	-8.3
		2	-2.0	-0.4	-11.0	6.1	-8.2
		2	-3.7	-0.4	-10.5	8.5	-7.9
		2	-1.6	-0.4	-9.8	4.5	-7.7
		1	-1.5	-0.2	-13.5	9.5	-7.6

		2	-0.9	-0.8	-9.2	3.7	-7.2
		2	-1.7	-0.4	-11.3	7.7	-7.2
		2	-2.1	-0.3	-6.9	2.7	-6.8
		2	-2.6	-1.0	-12.2	12.2	-6.5
		2	0.1	-1.0	-12.8	9.2	-6.2
		2	-0.7	-1.0	-7.0	3.0	-5.8
		2	-3.3	-0.3	-1.8	0.1	-5.3
		2	-1.3	-0.2	-6.8	4.4	-4.7
		2	-1.0	-0.2	-5.3	1.9	-4.6
		1	-1.0	-0.2	-4.5	1.0	-4.4
		2	-0.7	-0.2	-3.2	0.3	-3.5
		2	-0.5	-0.1	-2.0	0.2	-2.4
		1	1.3	-0.5	-2.1	0.2	-0.7
Urotropine	C-H···N	8	-9.8	-2.6	-21.2	17.4	-19.9
		6	-4	-0.1	-11.9	10.3	-8.3
Cocrystal	O-H···O/π···π	1	-97.5	-22	-34.9	125.2	-72.4
	O-H···N	2	-78.8	-22	-25.6	107.3	-55.6
	O-H···N	2	-72.3	-16.8	-21.3	86	-54.3
	O-H···N	1	-78.2	-18.7	-19	97.2	-53.1
	π···π	1	-14.3	-1.4	-33	33.8	-24.1
	π···π	2	-6.1	-1.2	-27.5	13.1	-23.2
	C-H···O	2	-23.3	-3.7	-13.9	33.2	-19
	C-H···O	2	-12.2	-4.9	-12.6	16.9	-17
	C-H···N	2	-15.1	-3.1	-16.4	28.1	-15.2
		2	-8.9	-2.7	-6.2	7.9	-11.9
		2	-9.3	-1.9	-10	13.2	-11.8
		2	-7.6	-1.6	-13.3	14.9	-11.6
		2	-6.2	-1.3	-10.8	10.7	-10.3
		2	-3	-0.3	-11.8	7.7	-8.9
		1	-2	-0.5	-10.6	6.1	-8.1
		2	-4.9	-0.2	-13	14.5	-7.7
		2	-4	-1.3	-18	22.7	-6.9
		2	0.6	-0.5	-14.3	8.9	-6.8
		2	-1.5	-0.2	-6.1	0.9	-6.5
		2	-1.5	-0.2	-6.1	0.8	-6.5
		2	-0.5	-1	-11.8	8	-6.5
		2	-4.4	-0.8	-7	9.2	-5.7
		2	-2.9	-0.4	-1.6	0.1	-4.6
		1	2.2	-1.1	-2.9	0.2	-1

[a] times the interaction is present (*n*), electrostatic (E_E), polarization (E_P), dispersion (E_D), and exchange-repulsion (E_R). [b] $E_{tot} = k_E E_E + k_P E_P + k_D E_D + k_R E_R$, with *k* being scale factors.[2]

Energy framework diagrams

The energy framework diagrams for HKA, coformers and cocrystals are given below. Note that only the strongest pairwise interactions (< 15 kJ mol^{-1}) are shown and H-atoms are omitted for clarity.

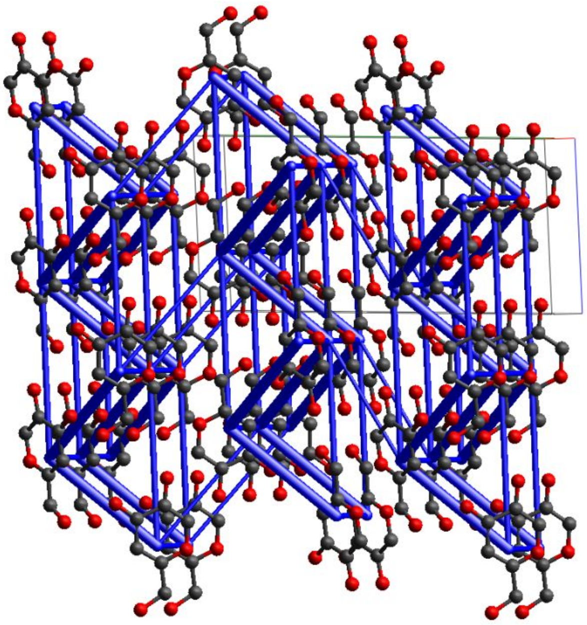

Figure S25. Energy framework diagram (total energy) for **HKA**. The energy scale factor is 60. Stabilizing contacts are shown in blue. The thickness corresponds to the strength. Pairwise interaction energies < 15 kJ mol^{-1} are omitted.

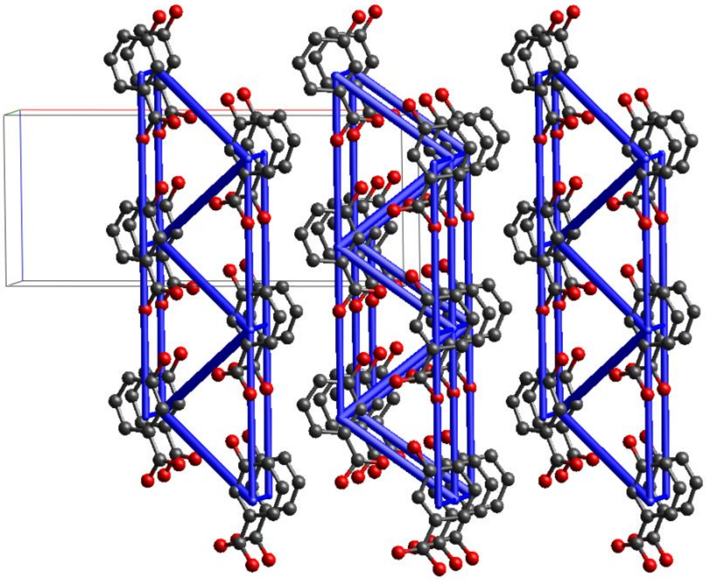

Figure S26. Energy framework diagram (total energy) for **3-HBA**. The energy scale factor is 60. Stabilizing contacts are shown in blue. The thickness corresponds to the strength. Pairwise interaction energies < 15 kJ mol^{-1} are omitted.

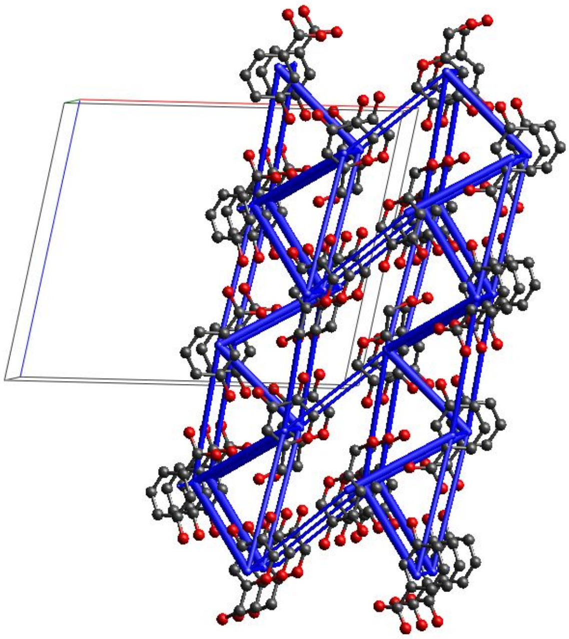

Figure S27. Energy framework diagram (total energy) for the **HKA 3-HBA co-crystal**. The energy scale factor is 60. Stabilizing contacts are shown in blue. The thickness corresponds to the strength. Pairwise interaction energies < 15 kJ mol^{-1} are omitted.

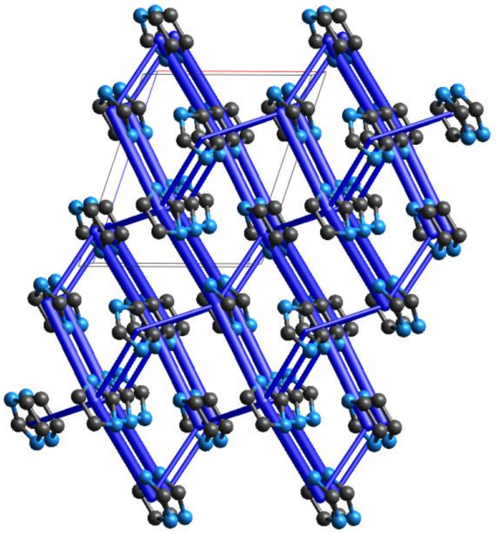

Figure S28. Energy framework diagram (total energy) for **imidazole** (alpha polymorph). The energy scale factor is 60. Stabilizing contacts are shown in blue. The thickness corresponds to the strength. Pairwise interaction energies < 15 kJ mol^{-1} are omitted.

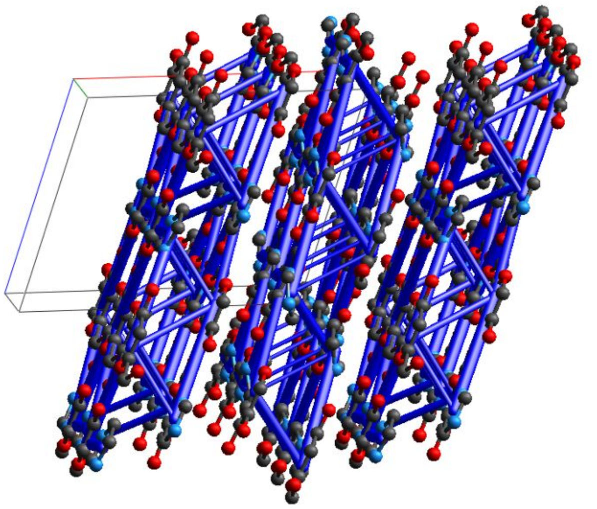

Figure S29. Energy framework diagram (total energy) for the **HKA imidazole co-crystal**. The energy scale factor is 60. Stabilizing contacts are shown in blue. The thickness corresponds to the strength. Pairwise interaction energies < 15 kJ mol^{-1} are omitted.

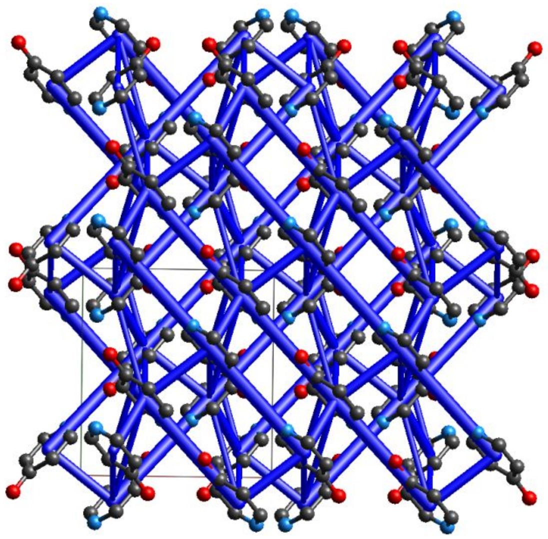

Figure S30. Energy framework diagram (total energy) for **pyridone**. The energy scale factor is 60. Stabilizing contacts are shown in blue. The thickness corresponds to the strength. Pairwise interaction energies < 15 kJ mol^{-1} are omitted.

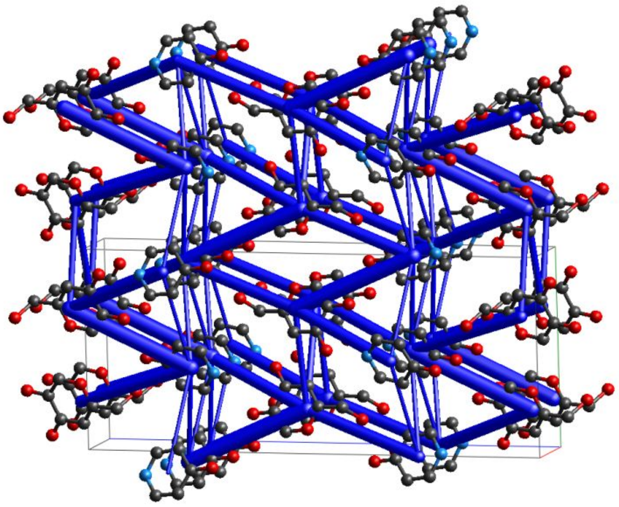

Figure S31. Energy framework diagram (total energy) for the **HKA pyridone co-crystal**. The energy scale factor is 60. Stabilizing contacts are shown in blue. The thickness corresponds to the strength. Pairwise interaction energies < 15 kJ mol^{-1} are omitted.

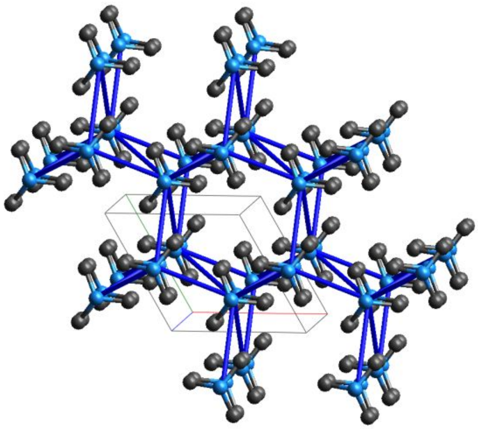

Figure S32. Energy framework diagram (total energy) for **DABCO**. The energy scale factor is 60. Stabilizing contacts are shown in blue. The thickness corresponds to the strength. Pairwise interaction energies < 15 kJ mol^{-1} are omitted.

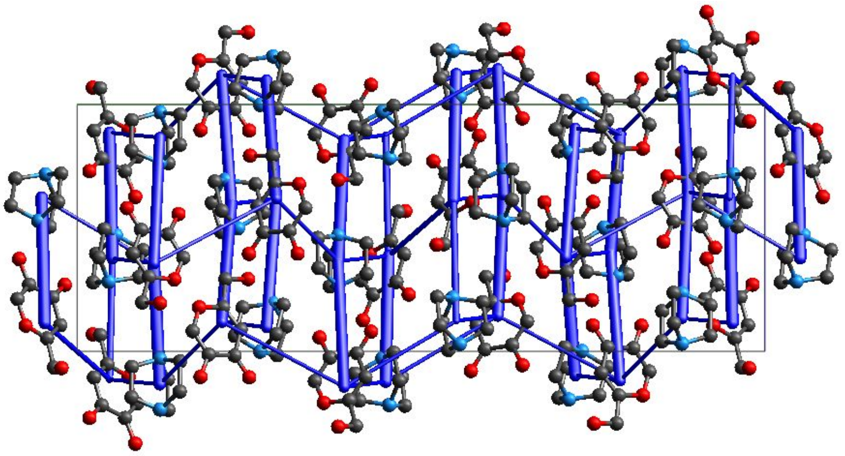

Figure S33. Energy framework diagram (total energy) for the **HKA DABCO co-crystal (orientation 1)**. The energy scale factor is 60. Stabilizing contacts are shown in blue. The thickness corresponds to the strength. Pairwise interaction energies < 15 kJ mol^{-1} are omitted.

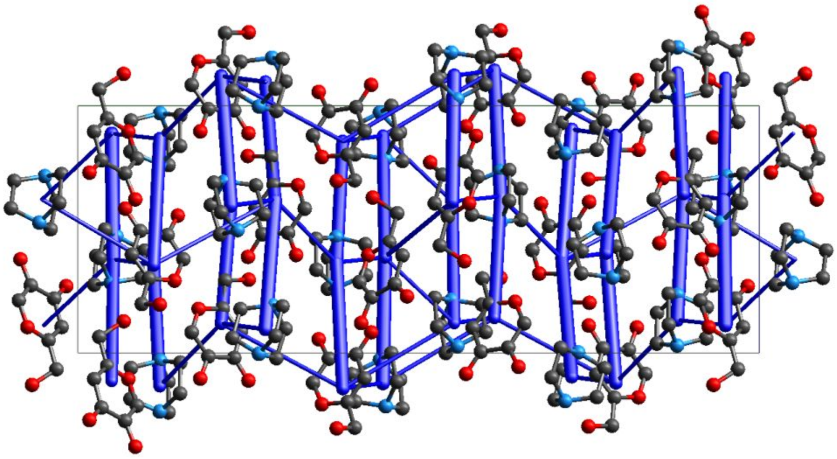

Figure S34 Energy framework diagram (total energy) for the **HKA DABCO co-crystal (orientation 2)**. The energy scale factor is 60. Stabilizing contacts are shown in blue. The thickness corresponds to the strength. Pairwise interaction energies < 15 kJ mol^{-1} are omitted.

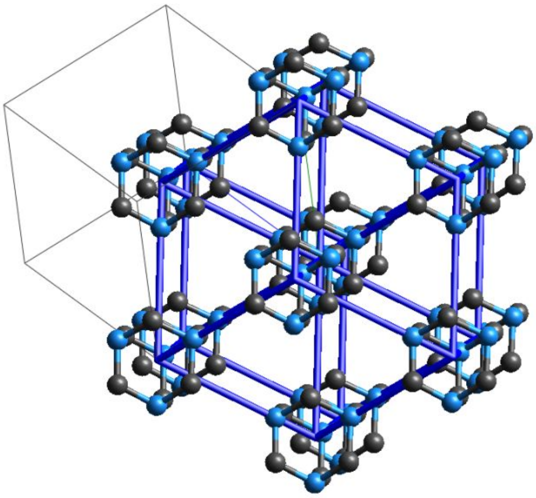

Figure S35. Energy framework diagram (total energy) for **urotropine**. The energy scale factor is 60. Stabilizing contacts are shown in blue. The thickness corresponds to the strength. Pairwise interaction energies < 15 kJ mol^{-1} are omitted.

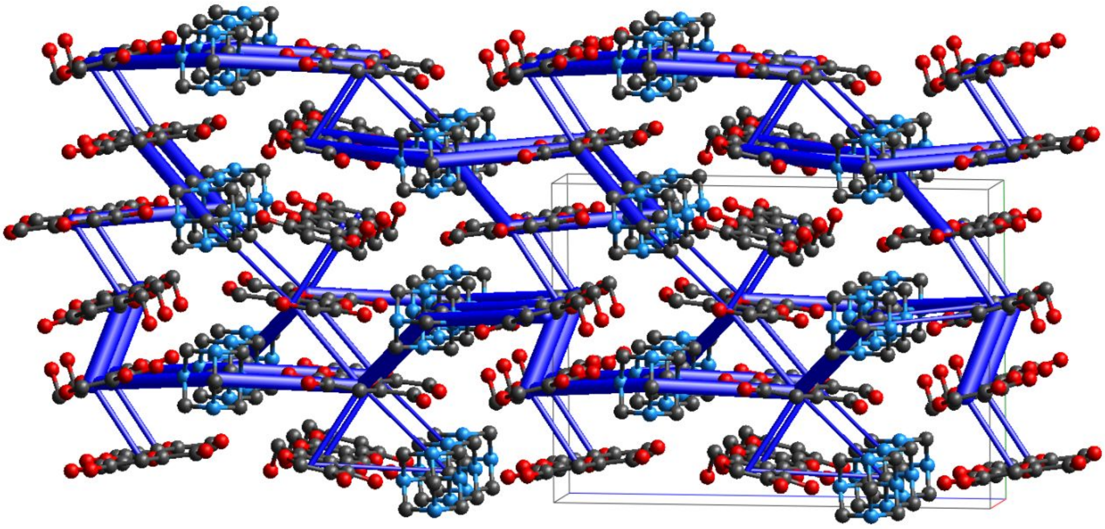

Figure S36. Energy framework diagram (total energy) for the **HKA urotropine co-crystal**. The energy scale factor is 60. Stabilizing contacts are shown in blue. The thickness corresponds to the strength. Pairwise interaction energies < 15 kJ mol^{-1} are omitted.

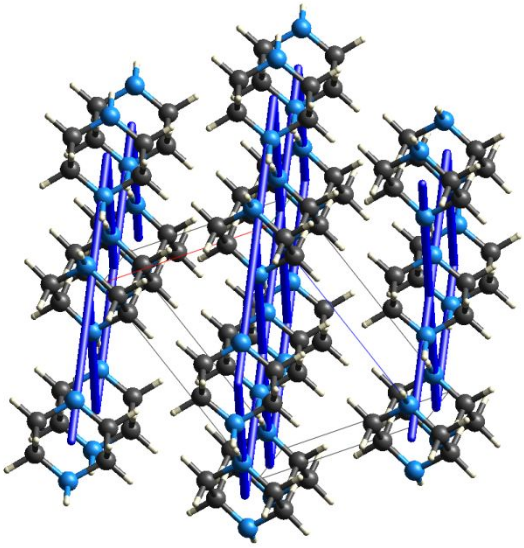

Figure S37. Energy framework diagram (total energy) for **piperazine**. The energy scale factor is 60. Stabilizing contacts are shown in blue. The thickness corresponds to the strength. Pairwise interaction energies < 15 kJ mol^{-1} are omitted.

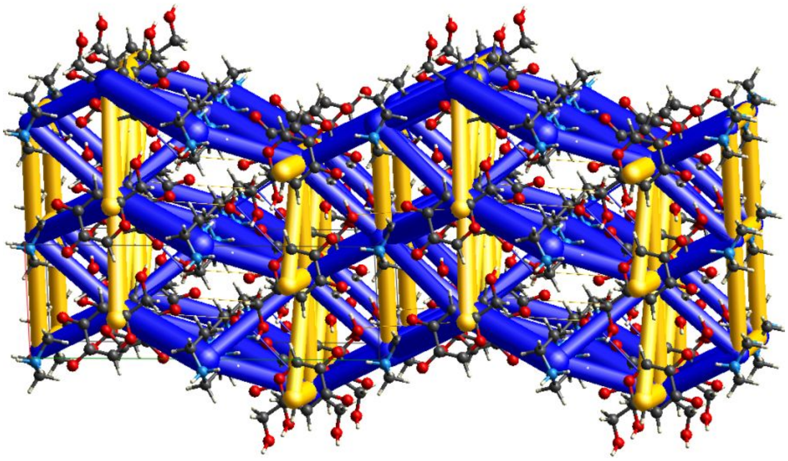

Figure S38. Energy framework diagram (total energy: blue – stabilizing, yellow - destabilizing) for **HKA piperazine co-crystal**. The energy scale factor is 6. The thickness corresponds to the strength. Pairwise interaction energies < 15 kJ mol^{-1} are omitted.

References

(1) Braun, D. E. The trimorphism of 3-hydroxybenzoic acid: an experimental and computational study. *CrystEngComm* **2021**, *23*, 2513-2519. DOI: https://doi.org/10.1039/D1CE00159K.

(2) Mackenzie, C. F.; Spackman, P. R.; Jayatilaka, D.; Spackman, M. A. CrystalExplorer model energies and energy frameworks: extension to metal coordination compounds, organic salts, solvates and open-shell systems. *IUCrJ* **2017**, *4*, 575-587. DOI: https://doi.org/10.1107/S205225251700848X.
